# Targeting Lymphotoxin Beta and Paired Box 5: a potential therapeutic strategy for soft tissue sarcoma metastasis

**DOI:** 10.1186/s12935-020-01632-x

**Published:** 2021-01-04

**Authors:** Runzhi Huang, Zhiwei Zeng, Penghui Yan, Huabin Yin, Xiaolong Zhu, Peng Hu, Juanwei Zhuang, Jiaju Li, Siqi Li, Dianwen Song, Tong Meng, Zongqiang Huang

**Affiliations:** 1 Department of Orthopedics, The First Affiliated Hospital of Zhengzhou University, 1 East Jianshe Road, Zhengzhou, 450052 China; 2 Division of Spine, Department of Orthopedics, Tongji Hospital Affiliated to Tongji University School of Medicine, 389 Xincun Road, Shanghai, China; 3 Department of Orthopedics, Shanghai General Hospital, School of Medicine, Shanghai Jiaotong University, 100 Haining Road, Shanghai, China; 4 Tongji University School of Medicine, 1239 Siping Road, Shanghai, 200092 China

**Keywords:** Soft tissue sarcoma, Transcription factor, Immune gene, Tumor-infiltrating immune cells, Metastasis

## Abstract

**Background:**

Soft tissue sarcomas (STS) has a high rate of early metastasis. In this study, we aimed to uncover the potential metastasis mechanisms and related signaling pathways in STS with differentially expressed genes and tumor-infiltrating cells.

**Methods:**

RNA-sequencing (RNA-seq) of 261 STS samples downloaded from the Cancer Genome Atlas (TCGA) database were used to identify metastasis-related differentially expressed immune genes and transcription factors (TFs), whose relationship was constructed by Pearson correlation analysis. Metastasis-related prediction model was established based on the most significant immune genes. CIBERSORT algorithm was performed to identify significant immune cells co-expressed with key immune genes. The GSVA and GSEA were performed to identify prognosis-related KEGG pathways. Ultimately, we used the Pearson correlation analysis to explore the relationship among immune genes, immune cells, and KEGG pathways. Additionally, key genes and regulatory mechanisms were validated by single-cell RNA sequencing and ChIP sequencing data.

**Results:**

A total of 204 immune genes and 12 TFs, were identified. The prediction model achieved a satisfactory effectiveness in distant metastasis with the Area Under Curve (AUC) of 0.808. LTB was significantly correlated with PAX5 (P < 0.001, R = 0.829) and hematopoietic cell lineage pathway (P < 0.001, R = 0.375). The transcriptional regulatory pattern between PAX5 and LTB was validated by ChIP sequencing data.

**Conclusions:**

We hypothesized that down-regulated LTB (immune gene) modulated by PAX5 (TF) in STSs may have the capability of inducing cancer cell metastasis in patients with STS.

## Background

Soft tissue sarcomas (STSs) are a group of rare and heterogeneous malignancies arising from resident cells of connective tissues that are comprised of more than 50 different histological subtypes and account for approximately 1% of all malignancies [1]. Despite advances in understanding STS tumorigenesis, management options have remained unchanged over the past few decades because of its rarity, complexity, late diagnosis and early metastasis [2]. In addition, due to the limited responsiveness to chemotherapy, surgery remains the standard treatment for patients with localized STS, but over 50% of patients may experience recurrence and metastasis after surgery [3]. Thus, novel treatments, such as targeted therapies, and the identification of biomarkers for identifying early metastatic disease are desperately needed.

Both molecular and cellular features have been shown to exert important influences on tumorigenesis and metastasis [4]. Transcription factors (TFs) are a group of proteins that regulate the transcription rate of genetic information from DNA to mRNA by binding to the specific DNA sequences. A large number of studies have indicated that TFs are actively involved in many human diseases, including cancers, in which they constitute approximately 20% of currently identified oncogenes [5]. Some abnormal biological behaviours, such as apoptosis, epithelial-mesenchymal transition (EMT), invasion, and metastasis, have also been attributed to the aberrant expression of TFs in various cancers [6, 7]. On the other hand, interactions and complicated communication among diverse tumour-infiltrating immune cells also plays a role in tumour metastasis and mortality prediction [8]. However, metastasis-related TFs and tumour-infiltrating immune cells in STS have not been explored and need to be further analysed.

In this study, we conducted a comprehensive analysis of TFs and immune gene profiling to examine the overall survival (OS) and metastasis-related TFs and immune genes in patients with STS and constructed a prognostic model. Then, we used the “Cell Type Identification by Estimating Relative Subsets of RNA Transcripts (CIBERSORT)” algorithm to detect tumour-infiltrating immune cells and their proportions in STSs. We also performed gene set enrichment analysis (GSEA), gene set variation analysis (GSVA) and Pearson correlation analysis to examine potential metastasis-related signalling pathways. Finally, we proposed an innovative and systematic hypothesis about aberrantly expressed TFs that regulate the expression of corresponding immune genes and promote STS metastasis, which may unveil significant and novel biomarkers and help to improve clinical management. Additionally, key genes and regulatory mechanisms were validated by single-cell RNA sequencing (scRNA-seq) and chromatin immunoprecipitation (ChIP-seq) data.

## Methods

### Data collection, differentially expressed genes (DEGs) and functional enrichment analysis

The Ethics Committee of the First Affiliated Hospital of Zhengzhou University approved this study. RNA sequencing profiles and clinical information of localized and metastatic STS samples were collected from the Cancer Genome Atlas (TCGA) database (https://tcgadata.nci.nih.gov/tcga/). Cancer-related transcription factors (TFs) were collected from the Cistrome Cancer database (http://cistrome.org/). Immune-related genes were retrieved from the ImmPort database (https://www.import.org/) and Molecular Signatures Database (MSigDB) v7.0 (https://www.gsea-msigdb.org/gsea/msigdb/index.jsp). HTseq-count and Fragments Per Kilobase of transcript per Million mapped reads (FPKM) profiles of 261 samples, including 121 localized STS and 55 metastatic STS samples, were assembled. “edgeR” was used to identify DEGs after removing non-STS-specific genes. Counts per million (CPM) and trimmed mean of M-values (TMM) algorithms were used for data normalization. Genes with a false discovery rate (FDR) P < 0.05 and log2(fold change) > 1 or < −1 were regarded as DEGs. Heatmaps and volcano plots were created to illustrate DEGs. Then, DEGs were analysed using Gene Ontology (GO) and Kyoto Encyclopedia of Genes and Genomes (KEGG) datasets to examine potential mechanisms of STS metastasis.

### Identification of OS-related immune genes

The expression of all immune-related genes and immune-related DEGs was extracted from previously downloaded RNA-seq profiles and the DEG list, respectively, and was used to generate a heatmap and volcano plot. Then, immune-related DEGs and clinical data were used in univariate Cox regression analysis to identify OS-related immune genes.

### Construction of a prognostic model based on OS-related immune genes

Based on the results of univariate Cox regression analysis, we extracted the most significant OS-related immune genes (P < 0.05 in univariate Cox regression analysis), all of which were included in multivariate Cox regression analysis to evaluate the significance of each OS-related immune gene with a β value (the regression coefficient of each integrated gene in the model). The risk score of No. i patient was calculated with the following formula:

$$ {\text{Risk score}}_{\text{i}} = \mathop \sum \limits_{a = 1}^{n} \beta {\text{a}} \times \left( {\text{expression level of gene a}} \right) $$

Then, individuals were divided into two risk groups based on the median risk score. The area under the ROC curve was analysed to assess the accuracy of the model. Kaplan–Meier survival analysis was used to compare the survival probability between the high- and low-risk groups. Individuals were reordered based on the risk score and a risk curve, survival state-related scatterplot, and heatmap of OS-related immune genes were plotted. Univariate and multivariate Cox regression analyses, modified by baseline information, were used to identify the independent prognostic value of the risk score, age, sex, race, and metastatic diagnosis (in multivariate Cox regression analysis, the variables were all corrected for demographics and clinical information, which also reduced the bias among individual patients).

### Identification of differentially expressed transcription factors

The expression of all the -related TFs and cancer-related DEGs was extracted from the previously downloaded RNA-seq profiles and DEG list, respectively, and was used to create a heatmap and volcano plot. Pearson correlation analysis was performed to examine the interaction and correlation between differentially expressed transcription factors and overall survival-related immune genes. Interaction pairs with correlation coefficients > 0.300 and P < 0.001 were included in the subsequent analysis.

### Identification of potential immune cell and KEGG pathway mechanisms

The quantity of 21 immune cell types in localized primary STS and metastatic samples was evaluated by CIBERSORT to further examine immune cells that drove metastasis. Then, correlation analysis was used to identify the correlation between immune cells and the biomarker, which was illustrated by a co-expression heatmap. Linear plots of biomarkers and immune cells with P < 0.001 were generated. Prognosis-related signalling pathways, identified by univariate Cox regression analysis based on gene set variation analysis (GSVA), were then subjected to correlation analysis with crucial metastasis-related biomarkers and illustrated by a co-expression heatmap. Metastasis-related signalling pathways were also identified by gene set enrichment analysis (GSEA). KEGG pathways in both GSEA and GSVA analysis are displayed by Venn plots. Then, linear plots were generated to show the correlation between the crucial biomarker and metastasis- and prognosis-related KEGG signalling pathways.

### Construction of a network with TFs, key biomarkers, immune cells, and KEGG pathways

To further discover the metastatic mechanisms in patients with STS, we constructed a network based on the interaction among prognosis-related and/or metastasis-related transcription factors, biomarkers, immune cells, and KEGG pathways with Cytoscape. Finally, the STS metastasis-related hypothesis based on bioinformatics was illustrated by a signalling diagram.

### Online database external validation

To obtain the complete annotation of selected TFs, key biomarkers, immune cells, and signalling pathways, multiple online databases were used to detect gene and protein expression levels, including cBioPortal [9, 10], GEPIA [11], K-M Plotter [12], PathCards [13], LinkedOmics [14], STRING [15], TISIDB [16], UALCAN [17] and CellMarkers [18].

### Immunohistochemistry (IHC) validation

Twenty-nine formalin-fixed paraffin-embedded (FFPE) tissue blocks from 29 sarcoma patients were deparaffinized and dehydrated. The slides were incubated overnight (4 °C) with an anti-PAX5 antibody (1:50 dilution, Proteintech), anti-LTB antibody (1:200 dilution, Bioworld), anti-CHSY1 antibody (1:100 dilution, Abcam), anti-CD19 antibody (1:50 dilution, Proteintech), anti-CD38 antibody (1:50 dilution, Proteintech), anti-CD138 (1:100 dilution, Cell Signalling Technology) and anti-SPM310 (1:100 dilution, Novus NBP2-34359) after routine rehydration, antigen retrieval, and blocking procedures. Next, all slides were labelled with polymer HRP for 30 min and haematoxylin as a counterstain for 5 min at room temperature.

Two pathologists examined the pathological sections and identified positive results when the cytoplasm of cancer cells was stained. The percentage score of tumour cells was as follows: negative (0), yellowish (1–4), light brown (5–8), and dark brown (9–12). The markers of B cells (CD19 and CD38) and plasma cells (CD138 (Syndean-1) [19] and SPM310 (Novus NBP2-34359)) were scored in the tumour and in the surrounding lymph nodes, respectively. In negative controls, the primary antibody was replaced by buffer. Additionally, correlation analysis and nonparametric tests (Mann–Whitney U test) were performed to evaluate the relationship between the IHC score and clinical features (grade of differentiation and metastasis during follow-up).

### Validation of the regulatory mechanism of transcription factors

Two algorithms (ENCODE Transcription Factor Targets and JASPAR) [20, 21] were used to re-predict the transcriptional regulatory pattern of LTB and PAX5 to further support our hypothesis. In addition, we conducted a comprehensive retrieval of a public database and found five ChIP-seq datasets for PAX5 (four from *Homo sapiens* and one from *Mus musculus*) [22–25]. Integrative Genomics Viewer (IGV) was used to normalize and visualize binding regions and peaks from different datasets [26].

### Validation of scRNA-seq data

The scRNA-seq data of the human alveolar rhabdomyosarcoma cell line Rh41 were downloaded from Gene Expression Omnibus (GEO) (GSE113660) to validate the distribution and expression of key genes [27, 28]. To integrate data analysis, the Seurat method was used [29]. During quality control, only genes expressed in more than 200 single cells and cells with transcript counts ranging from 1500 to 100,000 were integrated into further analysis. The “vst” method was utilized to identify variable genes. Then, principal component analysis (PCA) was performed based on variable genes, and jackstraw analysis was used to select the principal components (PCs) [29]. In terms of dimension reduction analysis, the UMAP (Uniform Manifold Approximation and Projection) method with a resolution of 0.50 was applied to identify cellular clusters based on the top 20 significant PCs [30]. DEGs were filtered when the absolute value of log2(FC) was > 0.5 and FDR was < 0.05 in each cluster. The distribution and expression of DEGs are illustrated in feature plots and violin plots, respectively. In addition, every cluster was annotated by the singleR method [31] and CellMarker database [18]. Moreover, the GSVA method was used to quantify the signalling pathway (50 hallmark pathways) activity in each single cell.

### Statistical analysis

All statistical analyses were performed with R version 3.5.1 (Institute for Statistics and Mathematics, Vienna, Austria; https://www.r-project.org). For descriptive statistics, the mean ± standard deviation was used for continuous variables with a normal distribution, while the median (range) was used for continuous variables with an abnormal distribution. Categorical variables are described by counts and percentages. Two-tailed P < 0.05 was regarded as statistically significant.

## Results

### Identification of DEGs and functional enrichment analysis

**Fig. 1:**
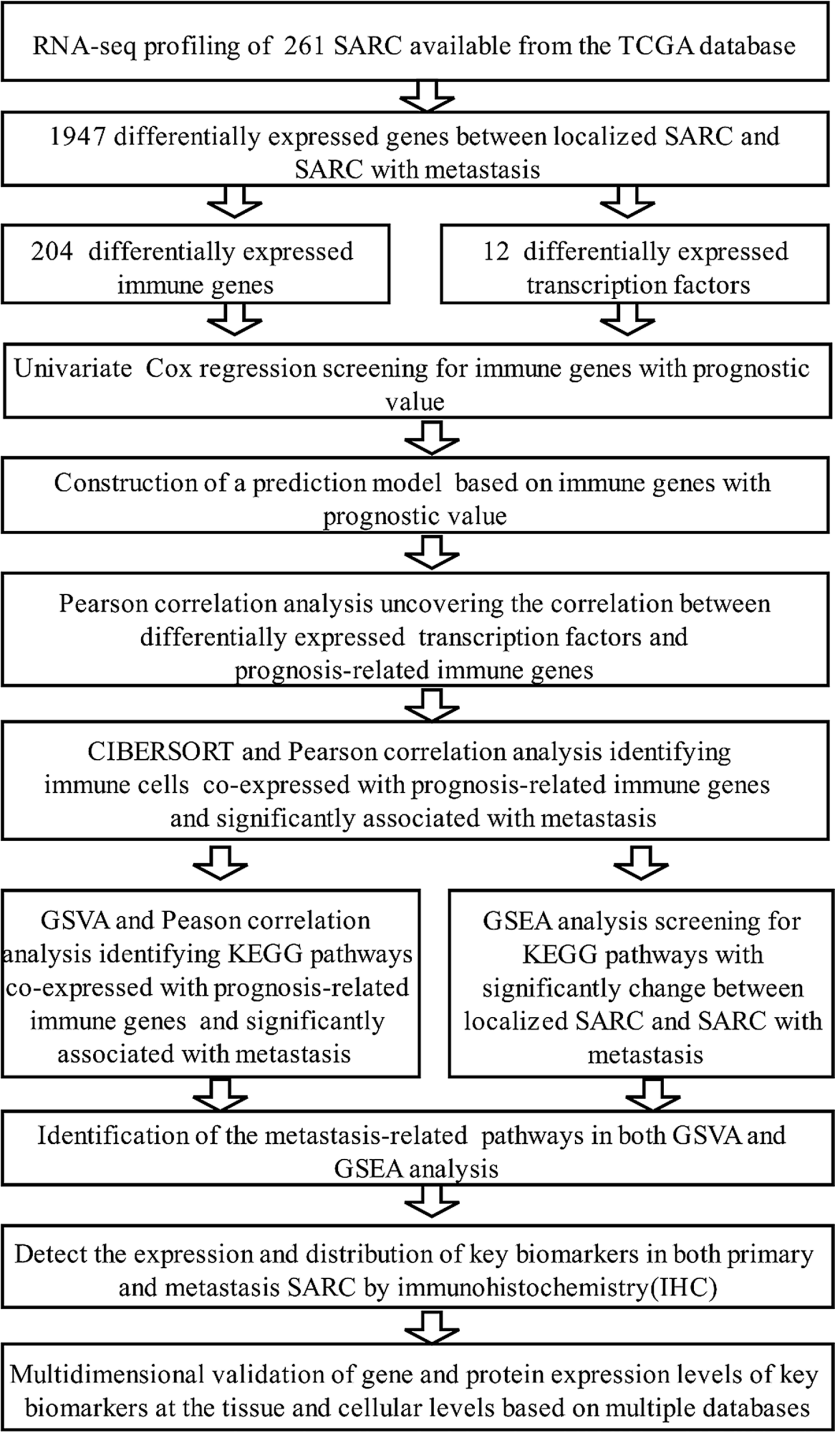
The analysis flowchart

**Fig. 2:**
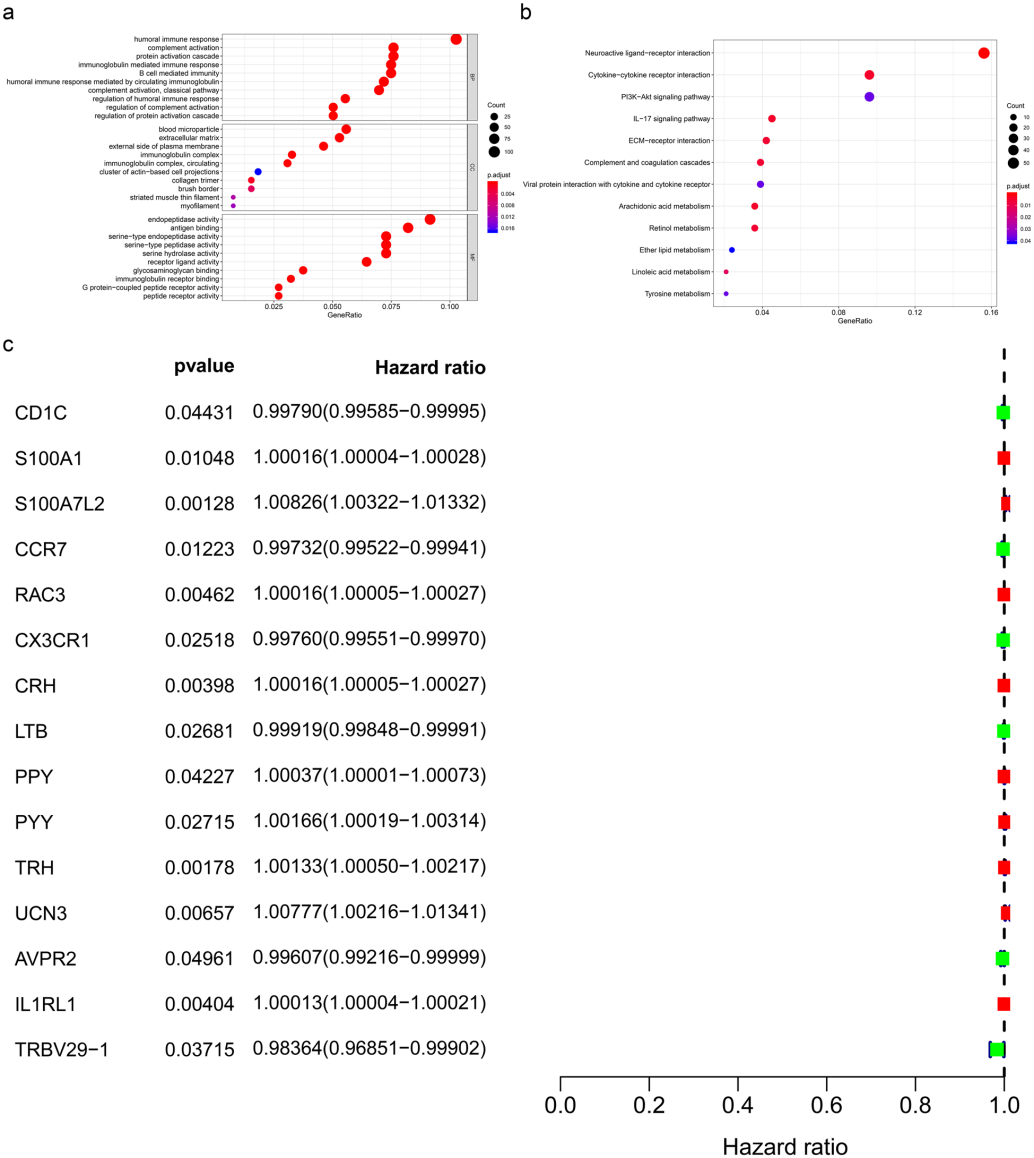
Functional enrichment analysis of significantly differentially expressed genes: GO (**a**) and KEGG (**b**) enrichment analysis of significantly differentially expressed genes. **c** The univariate Cox regression analysis for evaluating the prognostic value of identified immune genes. GO: Gene Ontology; KEGG: Kyoto Encyclopedia of Genes and Genomes; STS: soft tissue sarcoma

The analysis in this study is illustrated in Fig. 1. The baseline features of samples collected from the TCGA database are described in Additional file 1: Table S1. Genes with a log2(fold change) > 1 or < − 1 and FDR < 0.05 between localized STS and samples without metastasis were defined as DEGs. We identified 1947 differentially expressed genes (1375 down- and 572 upregulated), which is illustrated by a heatmap and volcano plot (Additional file 1: Figure S1A, B). To examine the potential mechanisms of the identified DEGs, GO and KEGG enrichment analyses were performed. Several immune response processes, such as “humoral immune response”, “complement activation”, and “immunoglobulin mediated immune response” in biological process (BP), “immunoglobulin complex” in cellular component (CC), and immune function, including “antigen binding” and “immunoglobulin receptor binding”, in molecular function (MF), were significantly different in GO analysis (Fig. 2a). KEGG enrichment analysis indicated that some key pathways, such as “cytokine–cytokine receptor interaction”, were significantly different between localized STS with and without metastasis (Fig. 2b).

### Identification of differentially expressed and prognosis-related immune genes

Differentially expressed immune genes (log2(fold change) > 1 or < − 1 and FDR < 0.05) are illustrated in the heatmap and volcano plot (Additional file 1: Figure S1C, D). To identify prognosis-related immune genes, univariate Cox regression analysis was performed, in which 6 protective factors and 9 risk factors were found. Among these factors, LTB (HR = 0.999, 95% CI (0.998–0.999), P = 0.027) was found to be inversely correlated with prognosis in patients with STS (Fig. 2c).

### Establishment of the prediction model

**Fig. 3:**
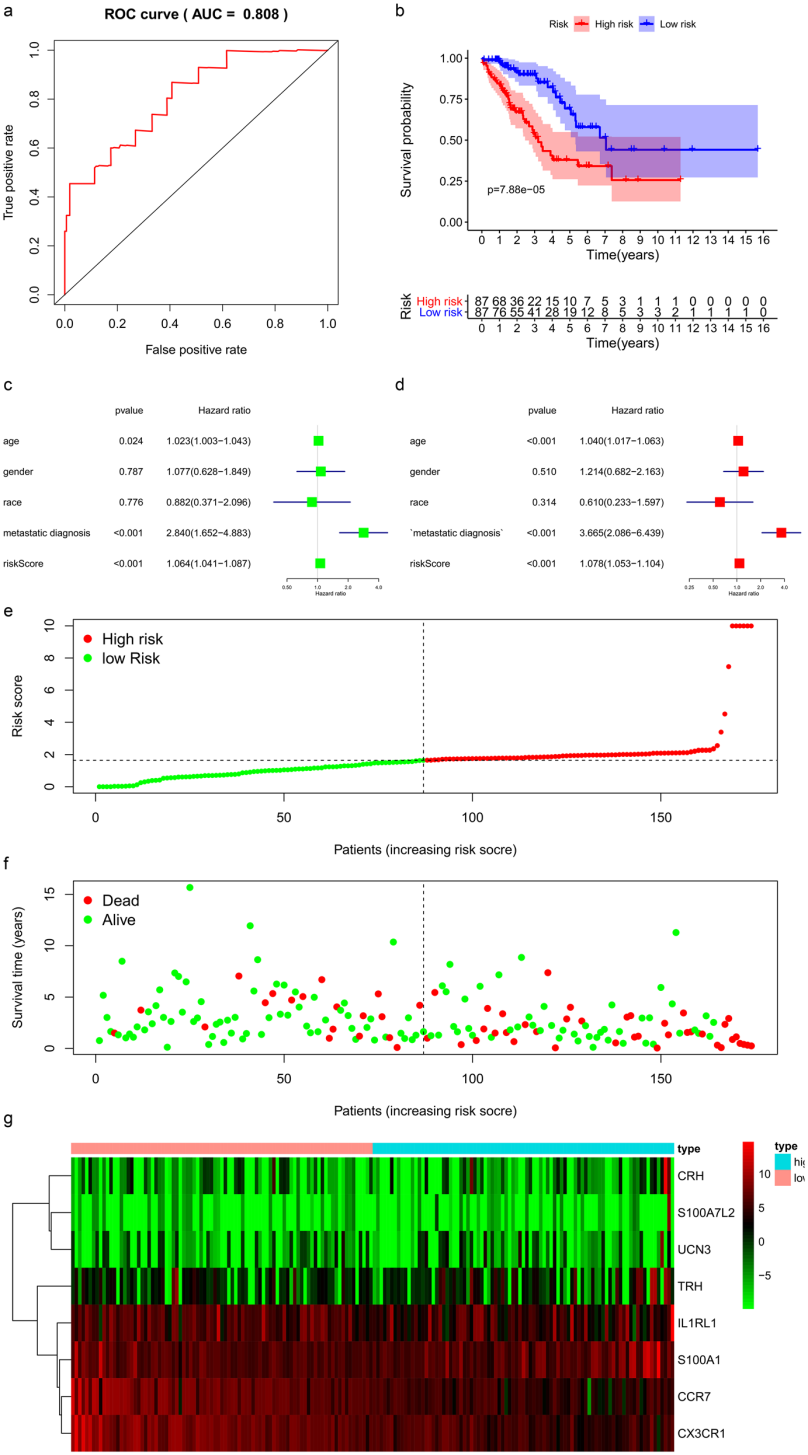
Prognostic model for STS patients: **a** The ROC curve for evaluating the accuracy of the prediction model. **b**The Kaplan–Meier analysis of the prediction model. **c** The univariate and multivariate Cox regression analysis of risk score, age, gender, race, and metastatic diagnosis for evaluating the independent prognostic value of the risk score. **d** The risk curve of each patient by risk score. **e** The scatter plot of the samples. The green and red dots representing survival and death, respectively. **f** The heatmap of immune genes screened by Lasso regression

Immune genes identified by univariate Cox regression analysis were included in Lasso regression analysis, and we found that key immune genes were significantly correlated with patient prognosis. Individuals were medially divided into the low- or high-risk group based on the risk score. The results indicated the good effectiveness of the prediction model with a high area under the curve (AUC) of the ROC curve (0.808) (Fig. 3a) and a significant difference in Kaplan–Meier analysis (P < 0.001) (Fig. 3b).

Risk curves and scatterplots were created to display the risk score and survival status of each patient with STS. Patients in the high-risk group had a higher mortality than those in the low-risk group (Fig. 3d, e). The heatmap shows the expression of CRH, S100A7L2, UCN3, TRH, IL1RL1, S100A1, CCR7, and CX3CR1, which were all included in the prognostic model (Fig. 3f).

To verify the independent prognostic value of the risk score and other clinical features, including age, sex, race, and metastatic diagnosis, both univariate and multivariate Cox regression analyses were performed. The risk score was proven to be an independent predictor in both univariate (HR = 1.064, 95% CI 1.041–1.087, P < 0.001) and multivariate Cox regression analyses (HR = 1.078, 95% CI 1.053–1.104, P < 0.001) (Fig. 3c).

### PAX5 regulated LTB to promote STS metastasis

Differentially expressed TFs (log2(fold change) > 1 or < −1 and FDR < 0.05) are illustrated in the heatmap and volcano plot (Additional file 1: Figure S1C). To explore the relationship between the identified TFs and key immune genes, Pearson correlation analysis was performed. Only regulation pairs with correlation coefficients < −0.300 or > 0.300 and P < 0.001 were selected to construct the regulatory network in subsequent analysis. We found that ASCL1-TPBV29-1 (P < 0.001, R = 0.36), PAX5-CD1C (P < 0.001, R = 0.31), PAX5-CCR7 (P < 0.001, R = 0.67), PAX5-LTB (P < 0.001, R = 0.829), and TFAP2A-S100A7L2 (P < −0.001, R = 0.361) were 5 pairs that met the screening threshold. Moreover, the PAX-LTB interaction had the greatest correlation coefficient among the pairs; thus, we entered that pair into the subsequent analysis (Figure S2).

### Molecular and signalling pathway mechanisms of LTB triggering STS metastasis

**Fig. 4:**
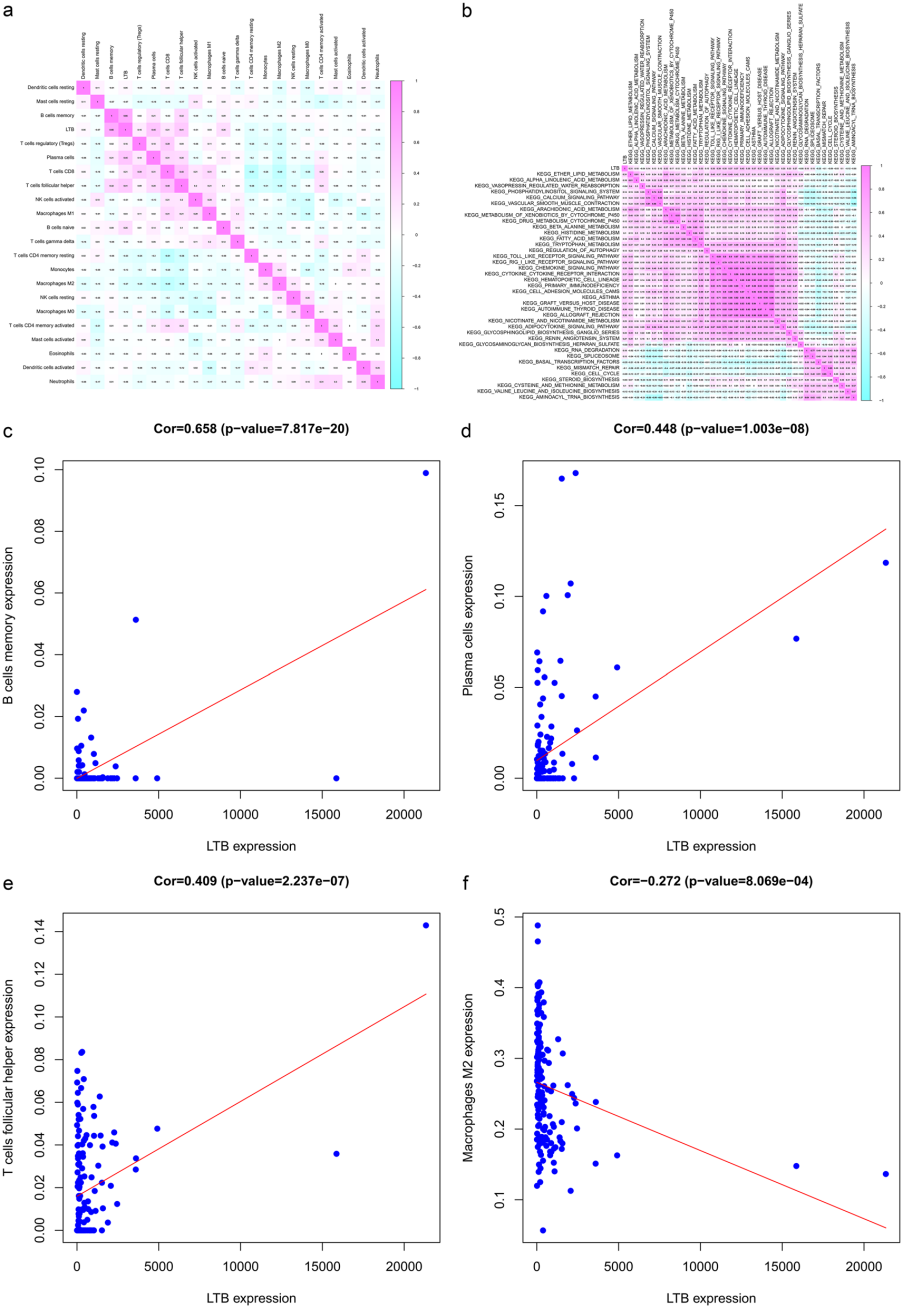
Identification of cytological and signaling pathways mechanisms in STS metastasis: **a** Co-expression heatmap between LTB and 21 immune cells. **b** Co-expression heatmap between LTB and prognosis-related KEGG pathways screened by GSVA and univariate Cox regression analysis. **c**–**f** Correlation relationship between LTB and immune cells

To further explore the potential cellular and signalling pathway mechanisms, CIBERSORT and GSVA were performed, in which the quantity of 21 immune cell types was evaluated, and 39 signalling pathways related to prognosis in patients with STS were screened. Pearson correlation analysis was applied to examine the correlation among LTB, immune cells, and prognosis-related KEGG pathways (Fig. 4a, b). The results showed that LTB was significantly correlated with B cell memory expression (P < 0.001, R = 0.658), plasma cells (P < 0.001, R = 0.448), follicular helper T cells (P < 0.001, R = 0.409), and M2 macrophages (P < 0.001, P = −0.272) (Fig. 4c–f).

### Identification of signalling pathways

**Fig. 5:**
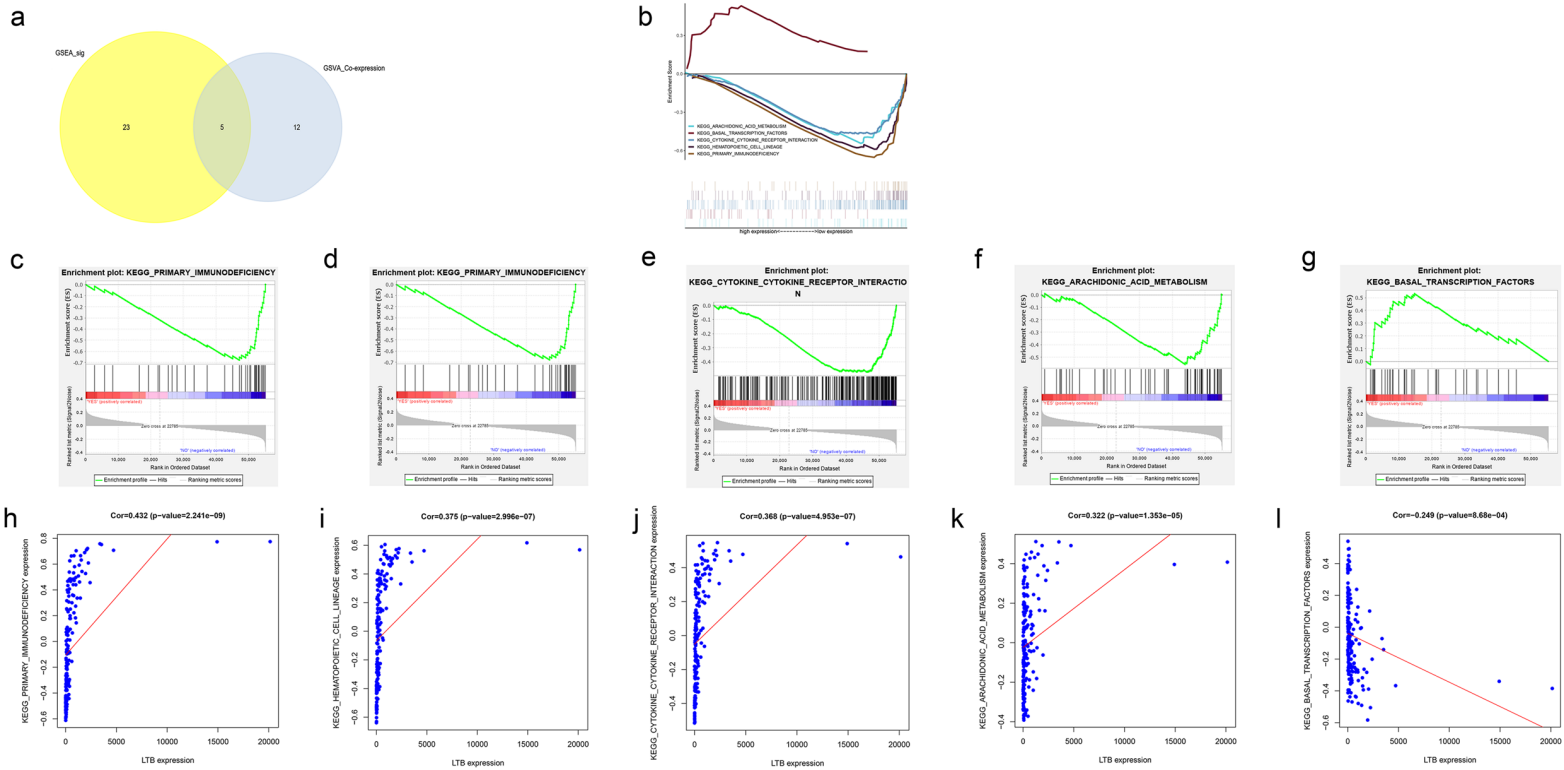
Further discovering of potential signaling pathways underlying STS metastasis: **a** Venn plot illustrating the number of KEGG pathways related to STS metastasis in GSEA and GSVA. **b** Integrated plot showing the GSEA analysis. **c**–**g** Significant KEGG pathways identified by GSEA analysis. **h**–**l** Results of Pearson correlation analysis between LTB and key KEGG pathways

**Fig. 6:**
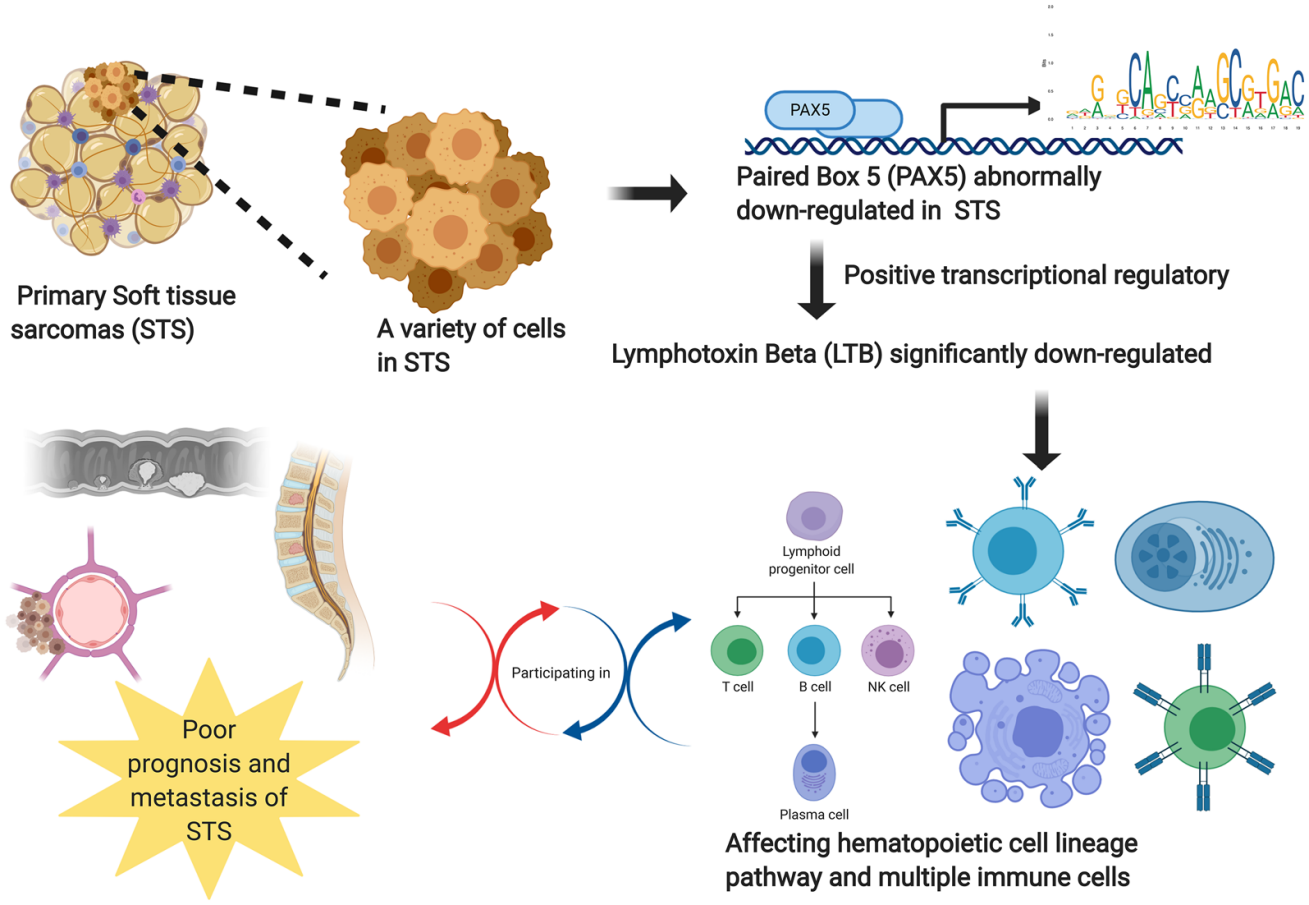
The illustration of our scientific hypothesis

To further identify metastasis- and prognosis-related KEGG pathways, GSEA was also performed. The results showed that 5 key KEGG pathways were significant in both GSEA and GSVA, including the arachidonic acid metabolism pathway, basal transcription factor pathway, cytokine–cytokine receptor interaction pathway, haematopoietic cell lineage pathway, and primary immunodeficiency pathway (Fig. 5a–g). Pearson correlation analysis showed that LTB was significantly correlated with the primary immunodeficiency pathway (P < 0.001, R = 0.432) (Fig. 5h), haematopoietic cell lineage pathway (P < 0.001, R = 0.375) (Fig. 5i), cytokine–cytokine receptor interaction pathway (P < 0.001, R = 0.369) (Fig. 5j), arachidonic acid metabolism pathway (P < 0.001, R = 0.322) (Fig. 5k), and basal transcription factor pathway (P < 0.001, R = −0.249) (Fig. 5l). Our hypothesis regarding STS metastasis mechanisms is illustrated in Fig. 6.

### External validation with multiple online databases

To reduce the bias induced by pure bioinformatics analysis, we used multiple online databases to further prove the reliability of our study. First, we used the CellMarker and PathCards databases to explore the biomarkers of plasma cells (IL1A, IL5RA, and IL7) and haematopoietic cell lineage pathways (IL5RA, LY9, SLAMF7, and ICAM1), respectively. The Oncomine database showed that LTB, LY9, SLAMF7, and ICAM1 were downregulated, while IL5RA was upregulated in different STS-related studies (Additional file 1: Figure S2). UALCAN, K-M Plotter, TISIDB, and LinkedOmics revealed that LTB, IL1A, IL5RA, IL7, LY9, SLAMF7, and ICAM1 were all significantly correlated with STS patient prognosis (Additional file 1: Figure S3–S6). In addition, LTB, IL1A, LY9, and SLAMF7 were differentially expressed between normal and tumour tissues (Additional file 1: Figure S3). GEPIA showed that LTB, IL5RA, LY9, and ICAM1 were significantly correlated with prognosis (Additional file 1: Figure S7). To examine the relationship between LTB and other biomarkers, we conducted Spearman correlation analysis with different databases. In LinkedOmics, LTB was significantly correlated with PAX5 (P < 0.001, R = 0.31), IL1A (P = 0.008, R = 0.17), IL5RA (P < 0.001, R = 0.43), IL7 (P < 0.001, R = 0.51), LY9 (P < 0.001, R = 0.82), SLAMF7 (P < 0.001, R = 0.79), and ICAM1 (P < 0.001, R = 0.61) (Additional file 1: Figure S5). In GEPIA, LTB was significantly correlated with PAX5 (P < 0.001, R = 0.34), IL1A (P = 0.016, R = 0.15), IL5RA (P < 0.001, R = 0.41), IL7 (P < 0.001, R = 0.50), LY9 (P < 0.001, R = 0.81), SLAMF7 (P < 0.001, R = 0.79), and ICAM1 (P < 0.001, R = 0.56) (Additional file 1: Figure S7). In cBioPortal, LTB was significantly correlated with PAX5 (P < 0.001, R = 0.31), IL1A (P = 0.009, R = 0.16), IL5RA (P < 0.001, R = 0.43), IL7 (P < 0.001, R = 0.52), LY9 (P < 0.001, R = 0.83), SLAMF7 (P < 0.001, R = 0.79), and ICAM1 (P < 0.001, R = 0.60) (Additional file 1: Figure S8B-H). In addition, K-M survival analysis that integrated all the biomarkers in cBioPortal showed that the overall expression of biomarkers was significantly related to patient prognosis (Additional file 1: Figure S8I). Finally, the STRING database suggested that all the biomarkers were strongly connected with each other based on the protein–protein interaction network (Additional file 1: Figure S9). Table 1 summarizes the results of the external validation of biomarkers in SARC with multiple online databases. Additionally, we tried to apply two other algorithms (ENCODE Transcription Factor Targets and JASPAR) [20, 21] to re-predict the transcriptional regulatory pattern between LTB and PAX5 to further support our hypothesis, which suggested that the DNA binding domain of PAX was similar to the sequence of the promoter region of LTB (Fig. 6).

**Table 1:** External Validation of Biomarkers in SARC via Multiple Online Database

	PAX5	LTB (anti-oncogene)	IL1A (anti-oncogene)	IL5RA (anti-oncogene)	IL7 (anti-oncogene)	LY9 (anti-oncogene)	SLAMF7 (anti-oncogene)	ICAM1 (anti-oncogene)
Oncomine	Expression: NA	Expression: Low	Expression: NA	Expression: High	Expression: NA	Expression: Low	Expression: Low	Expression: Low
UALCAN	Expression: NA Survival: P = 0.290	Expression: P < 0.001 Survival: P = 0.006	Expression: P < 0.001 Survival: P = 0.019	Expression: P = 0.781 Survival: P = 0.031	Expression: 0.471 Survival: P = 0.041	Expression: P < 0.001 Survival: P = 0.008	Expression: P < 0.001 Survival: P = 0.013	Expression: P = 0.521 Survival: P = 0.030
K-M Plotter	Survival: P = 0.084	Survival: P < 0.001	Survival: P = 0.003	Survival: P < 0.001	Survival: P = 0.010	Survival: P < 0.001	Survival: P = 0.006	Survival: P = 0.015
GEPIA	Survival: P = 0.380 Correlation: P < 0.001 R = 0.34	Survival: P = 0.004	Survival: P = 0.056 Correlation: P = 0.016 R = 0.15	Survival: P < 0.001 Correlation: P < 0.001 R = 0.41	Survival: P = 0.120 Correlation: P < 0.001 R = 0.50	Survival: P = 0.006 Correlation: P < 0.001 R = 0.81	Survival: P = 0.078 Correlation: P < 0.001 R = 0.79	Survival: P = 0.037 Correlation: P < 0.001 R = 0.56
LinkedOmics	Survival: P = 0.500 Correlation: P < 0.001 R = 0.31	Survival: P < 0.001	Survival: P = 0.015 Correlation: P = 0.008 R = 0.17	Survival: P = 0.030 Correlation: P < 0.001 R = 0.43	Survival: P = 0.022 Correlation: P < 0.001 R = 0.51	Survival: P = 0.001 Correlation: P < 0.001 R = 0.82	Survival: P = 0.008 Correlation: P < 0.001 R = 0.79	Survival: P = 0.035 Correlation: P < 0.001 R = 0.61
TISIDB	Survival: NA	Survival: P = 0.006	Survival: P = 0.019	Survival: NA	Survival: P = 0.021	Survival: P < 0.001	Survival: P = 0.006	Survival: P = 0.043
cBioportal	Correlation: P < 0.001 R = 0.31		Correlation: P = 0.009 R = 0.16	Correlation: P < 0.001 R = 0.43	Correlation: P < 0.001 R = 0.52	Correlation: P < 0.001 R = 0.83	Correlation: P < 0.001 R = 0.79	Correlation: P < 0.001 R = 0.60

PAX5: Paired Box 5; LTB: Lymphotoxin Beta; IL1A: Interleukin 1 Alpha; IL5RA: Interleukin 5 Receptor Subunit Alpha; IL7: Interleukin 7; LY9: Lymphocyte Antigen 9; SLAMF7: Signaling Lymphocytic Activation Molecule Family Member 7; ICAM1: Intercellular Adhesion Molecule 1

### Immunohistochemistry (IHC)

**Fig. 7:**
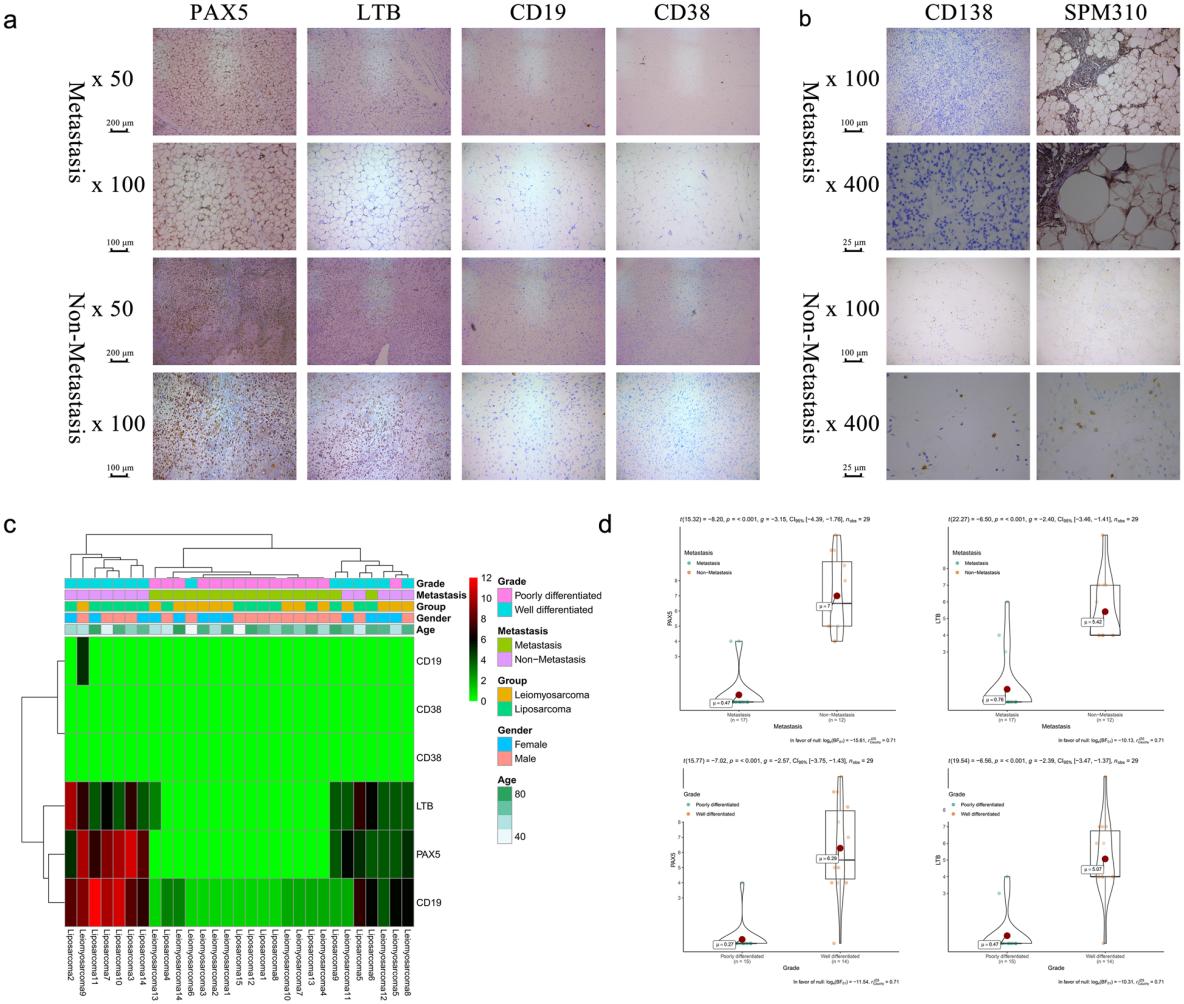
The results of immunohistochemistry (IHC). In total of 29 patients, 15 were diagnosed with liposarcoma (Metastasis occurred in eight patients during follow-up) and 14 were diagnosed with leiomyosarcoma (Metastasis occurred in nine patients during follow-up). Proteins of PAX5 and LTB were shown to be significantly down-regulated in the tumor cells of primary sarcomas with metastasis while the markers of B cells (CD19 and CD 38) were not detected in almost all primary tumor (**a**). Although CD19 and CD 38 were found in lymph node, as the advice of a pathologist in our hospital, these were not surprising as B cells are abundant in lymph node. Therefore, the IHC results of CD19 and CD 38 did not prove or disprove the hypothesis. Furthermore, in the absence of a good CD19 and CD38 antibody, we considered CD138 (Syndean-1) and SPM310 plasma cell marker antibody (Novus NBP2-34359) as other proven plasma cell markers. However, only four of 29 sarcomas were found plasma cells in the HE staining section of the tumor and none of the four patients had metastases (**b**). This might be because tumor-infiltrating immune cells tended to be located around the tumor rather than within it, and sarcomas tended to be excised en bloc, so there were no paracancer tissues as a control. Fortunately, the results of Mann–Whitney U test suggested that PAX5 (P < 0.001), LTB (P < 0.001) in were all highly expressed in well differentiated primary sarcomas and primary sarcomas without metastasis (**c**, **d**)

Among 29 patients, 15 were diagnosed with liposarcoma (metastasis occurred in eight patients during follow-up), and 14 were diagnosed with leiomyosarcoma (metastasis occurred in nine patients during follow-up). PAX5 and LTB proteins were significantly downregulated in the tumour cells of primary sarcomas with metastasis, while markers of B cells (CD19 and CD38) were not detected in almost all primary tumours (Fig. 7a). Although CD19 and CD38 were found in lymph nodes, as noted by a pathologist at our hospital, these findings were not surprising, as B cells are abundant in lymph nodes. Therefore, the IHC results of CD19 and CD38 did not prove or disprove the hypothesis that plasma cells were downstream of LTB. However, PAX5 and LTB proteins were shown to be significantly downregulated in the tumour cells of primary sarcomas with metastasis. Furthermore, in the absence of a good CD19 and CD38 antibody, we used anti-CD138 (Syndean-1) and anti-SPM310 antibodies as antibodies for plasma cell marker detection (Novus NBP2-34359), as these were other proven plasma cell markers. However, only four of 29 sarcomas were found to have plasma cells in HE staining of the tumour, and none of these four patients had metastases (Fig. 7b). This might be because tumour-infiltrating immune cells tended to be located around the tumour rather than within it, and sarcomas tended to be excised en bloc, so there were no paracancerous tissues that could be used as a control. The results of the Mann–Whitney U test suggested that PAX5 (P < 0.001) and LTB (P < 0.001) were all highly expressed in well-differentiated primary sarcomas and primary sarcomas without metastasis (Fig. 7c, d).

### ChIP-seq validation

**Fig. 8:**
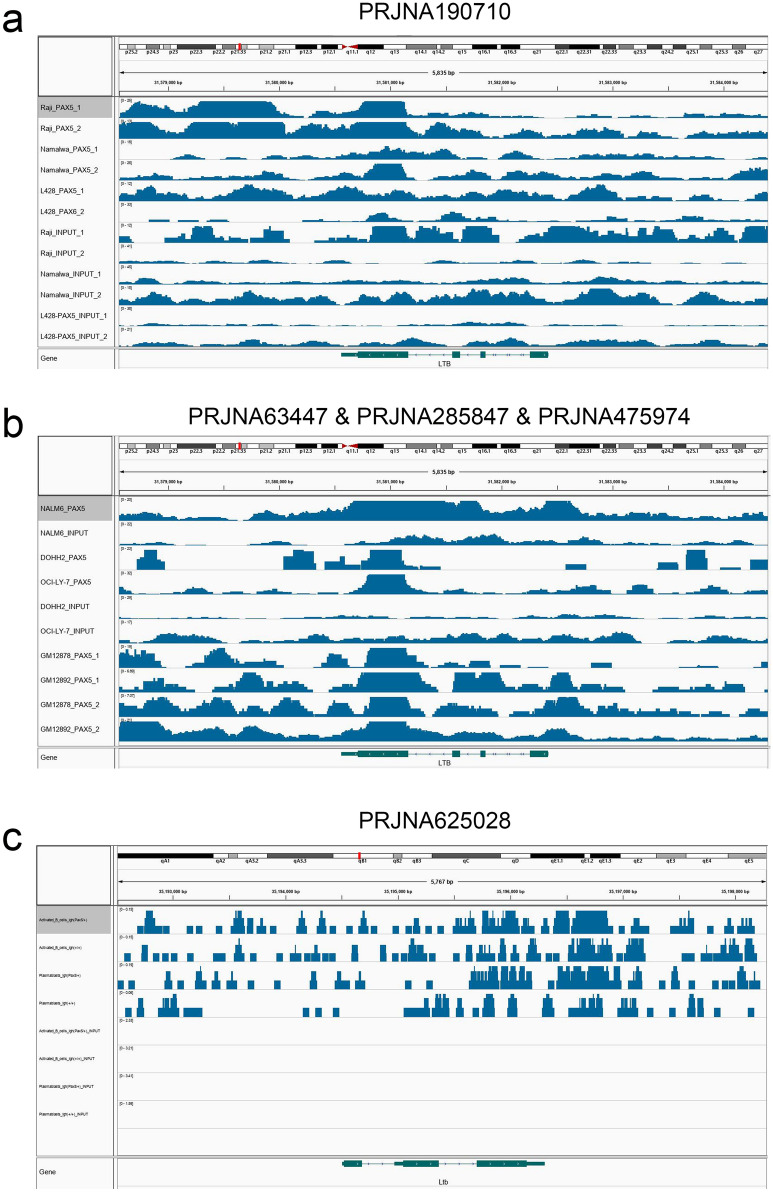
The results of ChIP-seq validation. In the three different kinds of B Lymphocyte in Hodgkin’s Lymphoma and Burkitt’s Lymphoma (Raji, Namalwa and L428 cells) (PRJNA190710), compared to the control group, the binding regions of PAX5 in LTB showed more bonding strength (input samples) (**a**). Similarly, more bonding strength of PAX5 and LTB was also illustrated in the NALM6, DOHH2, OCI-LY-7, GM1287 and GM12892 cells comparing to the control group (PRJNA63447, PRJNA285847 and PRJNA475974) (**b**). Besides, in Pax5 ChIP-seq data of activated B cells and plasmablasts (mus musculus), upregulated binding peaks were also found in Ltb sequences (PRJNA625028) (**c**). What’s more, binding peaks of Ltb were higher in activated B cells and plasmablasts from IghPax5/+ mice than the control group

A comprehensive retrieval of public databases (Sequence Read Archive (SRA), European Genome-phenome Archive (EGA) and The European Bioinformatics Institute (EBI)) was conducted, and five ChIP-seq datasets for PAX5 were filtered (four from *Homo sapiens* and one from *Mus musculus*) (Table S3) [22–25]. In the three different kinds of B lymphocytes in Hodgkin’s lymphoma and Burkitt’s lymphoma (Raji, Namalwa and L428 cells) (PRJNA190710), compared to that in cells in the control group, the binding regions of PAX5 in LTB showed higher binding strength (input samples) (Fig. 8a). Similarly, higher binding strength of PAX5 and LTB was also illustrated in NALM6, DOHH2, OCI-LY-7, GM1287 and GM12892 cells compared to that in cells in the control group (PRJNA63447, PRJNA285847 and PRJNA475974) (Fig. 8b). In addition, in Pax5 ChIP-seq data of activated B cells and plasmablasts (*Mus musculus*), upregulated binding peaks were also found in Ltb sequences (PRJNA625028) (Fig. 8c). Moreover, the binding peaks of Ltb were higher in activated B cells and plasmablasts from IghPax5/+ mice than in those from mice in the control group.

### Validation of scRNA-seq data

**Fig. 9:**
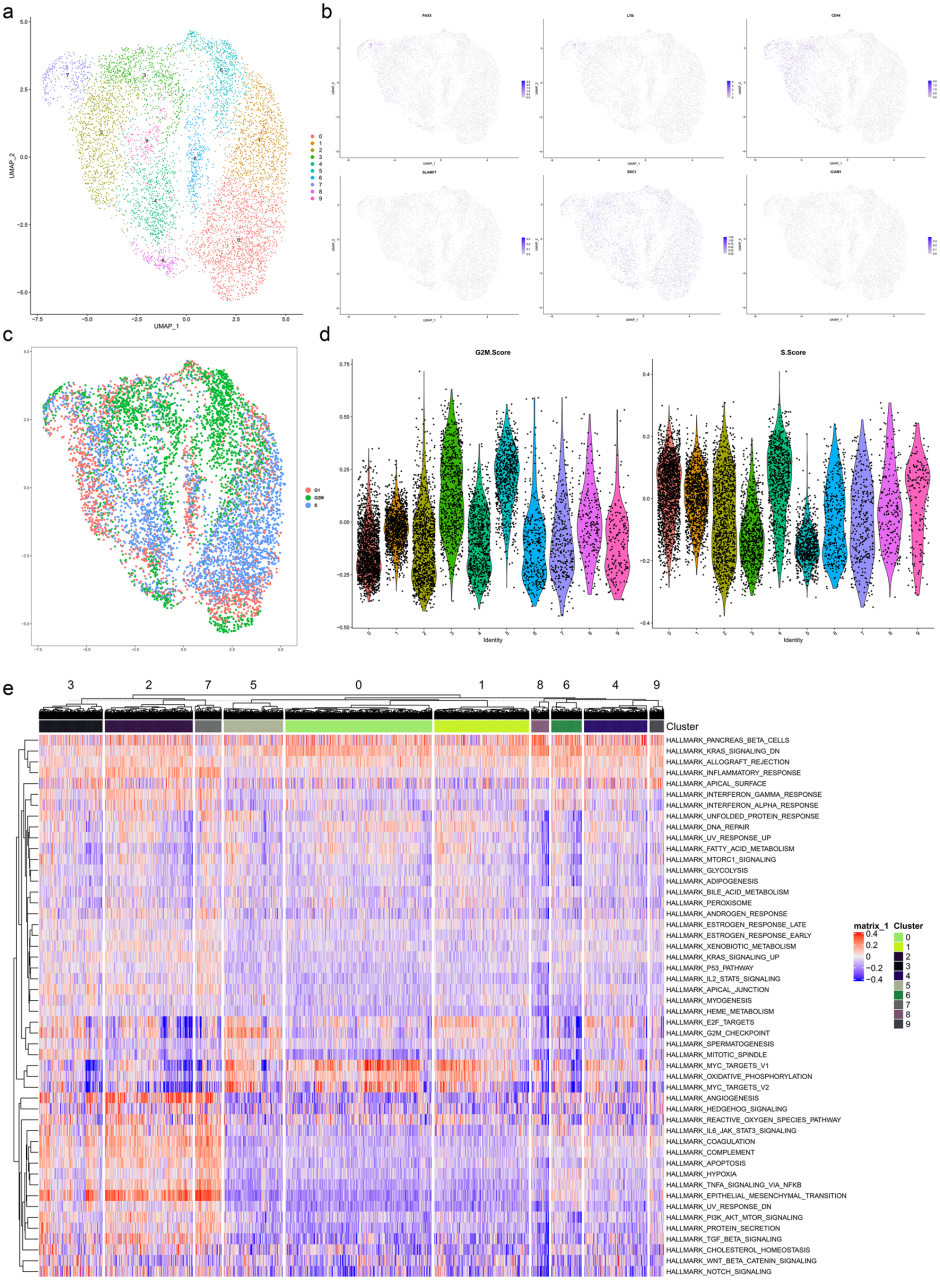
Validation of scRNA-seq data. The scRNA-seq data of the human alveolar rhabdomyosarcoma cell line Rh41 was downloaded from Gene expression omnibus (GEO) (GSE113660) to validate the distribution and expression of key genes (PAX5, LTB, IL1A, IL5RA, IL7, LY9, SLAMF7, SDC1 and ICAM1). Firstly, 7261 human alveolar rhabdomyosarcoma cells were reduced and clustered into ten cellular clusters by the UMAP method with a resolution of 0.50 (**a**). Except for PAX5, LTB and CD44 (Markers of stem cells) were significantly colocalization in the No. 7 clusters, SLAMF7, SDC1 (Syndecan-1) and ICAM1 were scattered among different clusters of rhabdomyosarcoma cells while IL1A, IL5RA, IL7 and LY9 were not detected in rhabdomyosarcoma cells (**b**). In cell cycle analysis, rhabdomyosarcoma cells with high expression of PAX5 and LTB were significantly located in the G2M and S phases (**c**–**d**). Moreover, GSVA heatmap demonstrated that some metastasis-related signaling pathways such as epithelial mesenchymal transformation (EMT) and angiogenesis were active in cells with high expression of PAX5 and LTB (E)

The scRNA-seq data of the human alveolar rhabdomyosarcoma cell line Rh41 were downloaded from Gene Expression Omnibus (GEO) (GSE113660) to validate the distribution and expression of key genes (PAX5, LTB, IL1A, IL5RA, IL7, LY9, SLAMF7, SDC1 and ICAM1). First, 7261 human alveolar rhabdomyosarcoma cells were reduced and clustered into ten cellular clusters by the UMAP method with a resolution of 0.50 (Fig. 9a). PAX5, LTB and CD44 (markers of stem cells) were significantly colocalized in the No. 7 cluster; SLAMF7, SDC1 (Syndecan-1) and ICAM1 were scattered among different clusters of rhabdomyosarcoma cells; while IL1A, IL5RA, IL7 and LY9 were not detected in rhabdomyosarcoma cells (Fig. 9b). In cell cycle analysis, rhabdomyosarcoma cells with high expression of PAX5 and LTB were significantly located in the G2M and S phases (Fig. 9c, d). Moreover, the GSVA heatmap demonstrated that some metastasis-related signalling pathways, such as epithelial-mesenchymal transition (EMT) and angiogenesis, were active in cells with high expression of PAX5 and LTB (Fig. 9e).

## Discussion

STSs, accounting for 1% of all malignancies, are difficult diagnosis early and accurately. In addition, effective management methods have not been established due to its rarity, histological heterogeneity, and diverse biological behaviours [32]. Moreover, STS is notorious for its high rate of wide, early metastasis [2]. Recently, many researchers reported that the aberrant expression of transcription factors, immune genes, and tumour-infiltrating immune cells played important roles in promoting multiple abnormal biological behaviours in tumour progression, including metastasis [33–35]. However, related mechanisms in STS have not yet been clearly explored. In this study, we identified 204 differentially expressed immune genes and 12 TFs. Based on 15 OS-related immune genes, we established a prediction model that was highly effective based on the K-M survival curve (P < 0.001) and ROC curve (AUC: 0.808). Based on the results of Pearson correlation analysis between TFs and immune genes, we found that LTB (an immune gene) was significantly correlated with PAX5 (a TF) (P < 0.001, R = 0.83). PAX5 and LTB proteins were shown to be significantly downregulated in the tumour cells of primary sarcomas with metastasis based on IHC. Compared to the control group, the binding regions of PAX5 in LTB showed higher binding strength in five different ChIP-seq datasets. Additionally, PAX5, LTB and CD44 (markers of stem cells) were significantly colocalized in the scRNA-seq data of the human alveolar rhabdomyosarcoma cell line Rh41. These results all suggested that PAX5 and LTB might be potential predictors and therapeutic targets for STS metastasis.

Paired Box 5 (PAX5) encodes a member of the PAX family and functions as a TF through a DNA-binding motif, which is also known as a paired box. Paired box transcription factors are vital regulators of early organ development and tissue differentiation, and alterations in their expression are considered catalysts in neoplastic transformation [36]. Previous studies revealed that as a B-lymphoid transcription factor, PAX5 was downregulated in over 80% of pre-B cell acute lymphoblastic leukaemia (ALL), and its downregulation in lymphoid neoplasms was associated with promoter hypermethylation and poor clinical outcomes [37, 38]. In addition, aberrantly expressed PAX5 contributed to the tumorigenesis and malignant progression of many other cancers. In gastric cancer, PAX5 functioned as a tumour suppressor via promoter hypermethylation and suppressed cell proliferation and apoptosis. In addition, PAX5 also constrained cell invasion and metastasis by inducing MTSS1 (MTSS I-BAR Domain Containing 1) and TIMP1 (Tissue Inhibitor of Metalloproteinase 1) and inhibiting MMP1 (Matrix Metallopeptidase 1) [39]. Moreover, in non-small cell lung cancer (NSCLC), mesothelioma and oesophageal cancer, the expression of PAX5 was also decreased [40, 41]. In this study, we also found that the downregulation of PAX5 in STS was significantly correlated with distant metastasis and poor prognosis, which was in accordance with previous studies.

As a member of the tumour necrosis factor (TNF) ligand superfamily, Lymphotoxin Beta (LTB) forms a heteromeric complex with LT-alpha by acting as the primary ligand of the LT-beta receptor [42]. Previously, its function and mechanism were mainly believed to be involved in inflammatory responses, such as immune cell interactions and cytokine secretion regulation [43, 44]. Recently, its role in tumorigenesis and tumour evolution has received attention. The upregulation of LTB and its downstream targets, CXCL10 and NF-κB, was associated with tumorigenesis in HCV-related hepatocellular carcinoma (HCC) [45]. Additionally, LTB also interacted with methylated epithelial growth factor receptor (EGFR) in head and neck squamous cell carcinoma (HNSCC) to induce cetuximab resistance, leading to unfavourable outcomes. In papillary thyroid carcinoma, upregulated LTB also triggered metastasis [46]. However, in this study, the favourable prognostic role of LTB was evidenced by univariate Cox regression analysis and multiple online databases that showed that the expression of LTB was negatively correlated with metastasis and prognosis, which may help uncover the novel mechanisms of LTB as a tumour suppressor in tumorigenesis and metastasis. However, all of the above studies on PAX5 and LTB were conducted in cancer cells rather than tumour-infiltrating immune cells (tumour-infiltrating B cells and plasma cells).

To identify immune cells that are actively involved in metastasis in tumour tissues, we conducted CIBERSORT and Pearson correlation analysis of LTB and key immune cells, the results of which suggested the importance of plasma cells (P < 0.001, R = 0.45). Multiple online databases were also used to test the prognostic values of the biomarkers of plasma cells, including IL1A, IL5RA, and IL7. Plasma cells are a group of terminally differentiated B cells originating from marginal zone or germinal centre B cells. As an indispensable component of the humoural immune system, plasma cells play an important role in immune protection by secreting clonospecific immunoglobulins. The differentiation, development, and function of plasma cells are regulated and influenced by a variety of cytokines and transcription factors [47]. In several cancers, the dense infiltration of plasma cells was associated with prolonged survival [48]. In mice with hepatocellular carcinoma (HCC), the depletion of plasma cells suppressed the growth of tumours by promoting the antitumour T cell immune response, and the upregulated plasma cells were associated with poor prognosis in HCC patients [49]. However, unlike the definitive roles of tumour-infiltrating CD8^+^ T cells in antitumour immunity, the roles of tumour-infiltrating B cells and plasma cells are still unclear and controversial. Thus, our study may provide another potential mechanism [50]. The IHC results of CD19, CD38, CD138 (Syndean-1) and SPM310 did not prove or disprove the hypothesis that plasma cells were downstream of LTB. Thus, based on the results of this study, we could determine the transcriptional regulatory pattern between LTB and PAX5 and their cellular colocalization in cancer cells, and whether this regulatory mechanism existed in tumour-infiltrating B cells and plasma cells needs to be further validated.

Furthermore, to uncover the deeper mechanism underlying STS metastasis, GSEA and GSVA were performed to find prognosis-related KEGG pathways, including the arachidonic acid metabolism pathway, basal transcription factor pathway, cytokine–cytokine receptor interaction pathway, haematopoietic cell lineage pathway, and primary immunodeficiency pathway. In multiple online databases, we found that IL5RA, LY9, SLAMF7, and ICAM1, biomarkers of the haematopoietic cell lineage pathway, were significantly correlated with metastasis and prognosis in patients with STS. The haematopoietic cell lineage pathway is a complex renewal and differentiation process of blood cells, in which haematopoietic stem cells (HSCs) differentiate into common lymphoid progenitors (CLPs) and common myeloid progenitors (CMPs), ultimately promoting the lymphoid lineage and the myeloid lineage, respectively [51, 52]. Defects in the haematopoietic cell lineage pathway reportedly contribute to malignant cell transformation [53, 54]. In addition, reductions in haematopoietic stem cells were also associated with leukaemic stem cell persistence and poor prognosis in acute myeloid leukaemia [55].

Although many methods were used to control the bias introduced by pure bioinformatics analysis, there were still some weaknesses in this study. First, STS patients identified in the TCGA database in this study were mainly from Western countries. Thus, whether this prediction model is applicable for Asian populations remains unknown. In addition, most conclusions were made based on several computational predictions and few direct experiments. Thus, the evidence that LTB directly regulates PAX5 in STSs is not extensive. However, we have been performing a series of experiments, including flow cytometry, ChIP-seq, and single-cell sequencing, to further support the hypothesis proposed in this study.

## Conclusions

In conclusion, we established a satisfactory prediction model for patients with STS. Based on comprehensive bioinformatics analysis and preliminary experiments, we hypothesized that excessively downregulated LTB (an immune gene) modulated by PAX5 (a TF) in STSs induced cancer cell metastasis in patients with STS by modifying the haematopoietic cell lineage pathway. The transcriptional regulatory pattern between LTB and PAX5 could be determined by a bioinformatics algorithm and public ChIP-seq data.

## Supplementary information

**Additional file 1. Table S1** The baseline information of patients with STS;** Table S2** The regulatory relationship between transcription factors and immune genes;** Table S3** The series information of ChIP-seq datasets;** Figure S1** Identification of differentially expressed genes : The heatmap and volcano plot of differentially expressed genes (A, B), immune genes (C, D), and transcription factors (E, F) between localized STS and STS with metastasis;** Figure S2** Oncomine database validation. TB (A), LY9 (C), SLAMF7 (D), and ICAM1 (E) were down regulated, while IL5RA (B) were up-regulated in different STS-related studies;** Figure S3** UALCAN database validation. LTB (P = 0.006) (A), IL1A (P = 0.019) (B), LY9 (P = 0.008) (C), SLAMF7 (P = 0.013) (D), IL5RA (P = 0.031) (E), IL7 (P = 0.041) (F), and ICAM1 (P = 0.030) (G) were significantly correlated with STS patients’ prognosis. Besides, expression of LTB (P < 0.001) (A), IL1A (P < 0.001) (B), LY9 (P < 0.001) (C), and SLAMF7 (P < 0.001) (D) were significantly different between normal and tumor tissues;** Figure S4** K-M Plotter database validation. LTB (P < 0.001) (A), IL1A (P = 0.003) (B), IL5RA (P < 0.001) (C), IL7 (P = 0.041) (D), LY9 (P < 0.001) (E), SLAMF7 (P = 0.006) (F), and ICAM1 (P = 0.015) (G) were significantly correlated with patients’ prognosis;** Figure S5** TISIDB database validation. LTB (P = 0.006) (A), IL1A (P = 0.019) (B), IL7 (P = 0.021) (C), LY9 (P < 0.001) (D), SLAMF7 (P = 0.006) (E), and ICAM1 (P = 0.043) (F) were significantly correlated with patients’ prognosis;** Figure S6** LinkedOmics database validation. LTB (P < 0.001) (A), IL1A (P < 0.015) (C), IL5RA (P < 0.030) (D), IL7 (P < 0.022) (E), LY9 (P = 0.001) (F), SLAMF7 (P = 0.008) (G), and ICAM1 (P = 0.035) (H) were significantly correlated with patients’ prognosis. LTB were significantly correlated with PAX5 (P < 0.001, R = 0.31) (B), IL1A (P = 0.008, R = 0.17) (C), IL5RA (P < 0.001, R = 0.43) (D), IL7 (P < 0.001, R = 0.51) (E), LY9 (P < 0.001, R = 0.82) (F), SLAMF7 (P < 0.001, R = 0.79) (G), and ICAM1 (P < 0.001, R = 0.61) (H). (I) Volcano plot and heatmaps displayed the genes most significantly correlated with LTB;** Figure S7** GEPIA database validation. LTB (P = 0.004) (A), IL5RA (P < 0.001) (B), LY9 (P = 0.006) (C), and ICAM1 (P = 0.037) (D) were significantly correlated with prognosis. LTB was significantly correlated with PAX5 (P < 0.001, R = 0.34) (E), IL1A (P = 0.016, R = 0.15) (F), IL5RA (P < 0.001, R = 0.41) (G), IL7 (P < 0.001, R = 0.50) (H), LY9 (P < 0.001, R = 0.81) (I), SLAMF7 (P < 0.001, R = 0.79) (J), and ICAM1 (P < 0.001, R = 0.56) (K);** Figure S8** cBioportal database validation. (A) mRNA expression of each biomarker illustrated by heatmap. Spearman correlation analysis shown that LTB was significantly correlated with PAX5 (P < 0.001, R = 0.31) (B), IL1A (P = 0.009, R = 0.31) (C), IL5RA (P < 0.001, R = 0.43) (D), IL7 (P < 0.001, R = 0.52) (E), LY9 (P < 0.001, R = 0.83) (F), SLAMF7 (P < 0.001, R = 0.79) (G), and ICAM1 (P < 0.001, R = 0.60) (H). (I) K-M survival analysis integrated with all the biomarkers shown that the overall expression of biomarkers was significantly related to patients’ prognosis;** Figure S9** Protein-protein interaction network. (A) PathCards database provided the main biomarkers actively involved in hematopoietic cell lineage pathway, including IL5RA, LY9, SLAMF7, and ICAM1. (B) STRING database shown that all the biomarkers were tightly connected with each other.

## Data Availability

The datasets generated and/or analysed during the current study are available in the Cancer Availability of data and materialsGenome Atlas (TCGA) database (https://tcgadata.nci.nih.gov/tcga/), the Cistrome Cancer database (http://cistrome.org/), the ImmPort database (https://www.import.org/). Multiple other database of external validation also support the findings of this study, including cBioportal (https://www.cbioportal.org/), GEPIA (http://gepia.cancer-pku.cn/), K-M Plotter (http://kmplot.com/), PathCards (http://pathcards.genecards.org/), LinkedOmics (http://www.linkedomics.org/), STRING (https://string-db.org/), TISIDB (http://cis.hku.hk/TISIDB/), UALCAN (http://ualcan.path.uab.edu/), CellMarkers (http://biocc.hrbmu.edu.cn/CellMarker/). Gene expression omnibus (GEO) (https://www.ncbi.nlm.nih.gov/geo/query/acc.cgi?acc=GSE113660). Sequence Read Archive (SRA) (https://www.ncbi.nlm.nih.gov/Traces/study/?acc=PRJNA190710) (https://www.ncbi.nlm.nih.gov/Traces/study/?acc=PRJNA63447) (https://www.ncbi.nlm.nih.gov/Traces/study/?acc=PRJNA285847) (https://www.ncbi.nlm.nih.gov/Traces/study/?acc=PRJNA475974) (https://www.ncbi.nlm.nih.gov/Traces/study/?acc=PRJNA625028).

## Abbreviations

STS: Soft tissue sarcoma
LTB: Lymphotoxin Beta
PAX5: Paired Box 5
TCGA: The Cancer Genome Atlas
TF: Transcription factor
CIBERSORT: Cell type identification by estimating relative subsets of RNA transcripts
GSVA: Gene set variation analysis
GSEA: Gene set enrichment analysis
KEGG: Kyoto Encyclopedia of Genes and Genomes
AUC: The Area Under Curve
EMT: Epithelial-mesenchymal transition
OS: Overall survival
DEG: Differentially expressed gene
MSigDB: Molecular Signatures Database
FPKM: Fragments per kilobase of exon per million reads mapped
FDR: False discovery rate
GO: Gene Ontology
BP: Biological process
CC: Cellular component
MF: Molecular function
ALL: Acute lymphoblastic leukemia
IL1A: Interleukin 1 Alpha
IL5RA: Interleukin 5 Receptor Subunit Alpha
IL7: Interleukin 7
LY9: Lymphocyte Antigen 9
SLAMF7: Signaling Lymphocytic Activation Molecule Family Member 7
ICAM1: Intercellular Adhesion Molecule 1
TIMP1: Tissue Inhibitor of Metalloproteinase
MMP1: Matrix Metallopeptidase 1
NSCLC: Non-small cell lung cancer
TNF: Tumor necrosis factor
EGFR: Epithelial growth factor receptor
HNSCC: Head and neck squamous cell carcinoma
HCC: Hepatocellular carcinoma
HSC: Hematopoietic stem cell
CLP: Differentiate into common lymphoid progenitors
CMP: Common myeloid progenitors
ASCL1: Achaete-Scute Family BHLH Transcription Factor 1
TFAP2A: Transcription Factor AP-2 Alpha
TRBV29-1: T Cell Receptor Beta Variable 29-1
CD1C: Clustering of Differentiation 1C
CCR7: C-C Motif Chemokine Receptor 7
S100A7L2: S100 Calcium Binding Protein A7 Like 2
