# Supplementary material for: Targeting Lymphotoxin Beta and Paired Box 5: a potential therapeutic strategy for soft tissue sarcoma metastasis
Source: Cancer Cell Int. 2021 Jan 4;21:3. doi: 10.1186/s12935-020-01632-x (PMC7784354; doi:10.1186/s12935-020-01632-x)
Supplement: Supplementary file 1 — Additional file 1. Table S1 The baseline information of patients with STS; Table S2 The regulatory relationship between transcription factors and immune genes; Table S3 The series information of ChIP-seq datasets; Figure S1 Identification of differentially expressed genes : The heatmap and volcano plot of differentially expressed genes (A, B), immune genes (C, D), and transcription factors (E, F) between localized STS and STS with metastasis; Figure S2 Oncomine database validation. TB (A), LY9 (C), SLAMF7 (D), and ICAM1 (E) were down regulated, while IL5RA (B) were up-regulated in different STS-related studies; Figure S3 UALCAN database validation. LTB (P = 0.006) (A), IL1A (P = 0.019) (B), LY9 (P = 0.008) (C), SLAMF7 (P = 0.013) (D), IL5RA (P = 0.031) (E), IL7 (P = 0.041) (F), and ICAM1 (P = 0.030) (G) were significantly correlated with STS patients’ prognosis. Besides, expression of LTB (P < 0.001) (A), IL1A (P < 0.001) (B), LY9 (P < 0.001) (C), and SLAMF7 (P < 0.001) (D) were significantly different between normal and tumor tissues; Figure S4 K-M Plotter database validation. LTB (P < 0.001) (A), IL1A (P = 0.003) (B), IL5RA (P < 0.001) (C), IL7 (P = 0.041) (D), LY9 (P < 0.001) (E), SLAMF7 (P = 0.006) (F), and ICAM1 (P = 0.015) (G) were significantly correlated with patients’ prognosis; Figure S5 TISIDB database validation. LTB (P = 0.006) (A), IL1A (P = 0.019) (B), IL7 (P = 0.021) (C), LY9 (P < 0.001) (D), SLAMF7 (P = 0.006) (E), and ICAM1 (P = 0.043) (F) were significantly correlated with patients’ prognosis; Figure S6 LinkedOmics database validation. LTB (P < 0.001) (A), IL1A (P < 0.015) (C), IL5RA (P < 0.030) (D), IL7 (P < 0.022) (E), LY9 (P = 0.001) (F), SLAMF7 (P = 0.008) (G), and ICAM1 (P = 0.035) (H) were significantly correlated with patients’ prognosis. LTB were significantly correlated with PAX5 (P < 0.001, R = 0.31) (B), IL1A (P = 0.008, R = 0.17) (C), IL5RA (P < 0.001, R = 0.43) (D), IL7 (P < 0.001, R = 0.51) (E), LY9 (P < 0.001, R = 0.82) (F), SLAMF7 [file 12935_2020_1632_MOESM1_ESM.docx]

**Supplementary Data**

**Table S1** The baseline information of patients with STS

| Variables | Total Samples (N = 261) |
| --- | --- |
| **Metastasis** |  |
| NO | 121 (46.36%) |
| YES | 55 (21.07%) |
| Unknown | 85 (32.56%) |
| **Recurrence** |  |
| NO | 144 (55.17%) |
| YES | 29 (11.11%) |
| Unknown | 88 (.33.71%) |
| **Futime** |  |
| Mean ± SD | 796.5 ±907.09 |
| Median (Range) | 716 |
| **Histological_type** |  |
| Leiomyosarcoma (LMS) | 105 (40.23%) |
| Dedifferentiated liposarcoma | 59 (22.61%) |
| Pleomorphic 'MFH' / Undifferentiated pleomorphic sarcoma | 29 (11.11%) |
| Myxofibrosarcoma | 25 (9.58%) |
| Undifferentiated Pleomorphic Sarcoma (UPS) | 21 (8.05%) |
| Malignant Peripheral Nerve Sheath Tumors | 9 (3.45%) |
| Synovial Sarcoma - Monophasic | 6 (2.30%) |
| Desmoid Tumor | 2 (0.77%) |
| Sarcoma; synovial; poorly differentiated | 2 (0.77%) |
| Synovial Sarcoma - Biphasic | 2 (0.77%) |
| Giant cell 'MFH' / Undifferentiated pleomorphic sarcoma with giant cells | 1 (0.38%) |

| Transcription Factor | Immune Gene | Correlation coefficient | P Value | Regulation |
| --- | --- | --- | --- | --- |
| ASCL1 | TRBV29-1 | 0.360 | < 0.001 | Positive |
| PAX5 | CD1C | 0.311 | < 0.001 | Positive |
| PAX5 | CCR7 | 0.667 | < 0.001 | Positive |
| PAX5 | LTB | 0.829 | < 0.001 | Positive |
| TFAP2A | S100A7L2 | 0.361 | < 0.001 | Positive |

**Table S2** The Regulatory Relationship Between Transcription Factors and Immune Genes

ASCL1, Achaete-Scute Family BHLH Transcription Factor 1; PAX5, Paired Box 5; TFAP2A, Transcription Factor AP-2 Alpha; TRBV29-1, T Cell Receptor Beta Variable 29-1; CD1C, Clustering of Differentiation 1C; CCR7, C-C Motif Chemokine Receptor 7; LTB, Lymphotoxin Beta; S100A7L2, S100 Calcium Binding Protein A7 Like 2

**Table S3** The series information of ChIP-seq datasets

| **GSE_ID** | **SRA_ID** | **Disease** | **Biological Sources** | **PMID** |
| --- | --- | --- | --- | --- |
| GSE44551 | PRJNA190710 | Hodgkin's Lymphoma and Burkitt's Lymphoma | B Lymphocyte | 23842424 |
| GSE32465 | PRJNA63447 | Normal | B Lymphocyte and Lymphoblastoid | 24076218 |
| GSE69558 | PRJNA285847 | B-cell Lymphoma | B Lymphocyte | 26229090 |
| GSE115764 | PRJNA475974 | Acute lymphoblastic leukemia | NALM6 cells | 32885175 |
| GSE148556 | PRJNA625028 | Normal | Plasma cells | NA |


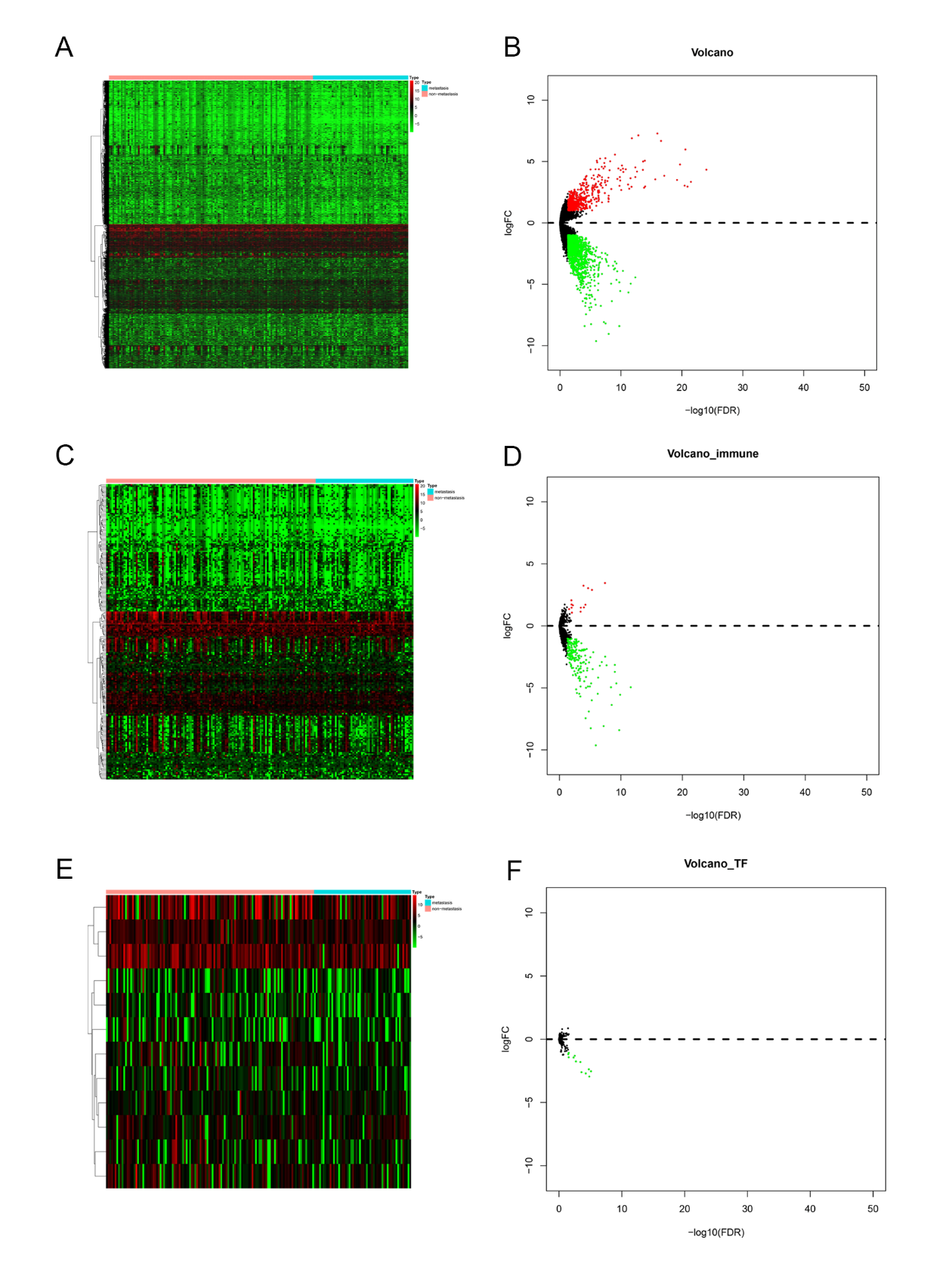


Figure S1 Identification of differentially expressed genes : The heatmap and volcano plot of differentially expressed genes (A, B), immune genes (C, D), and transcription factors (E, F) between localized STS and STS with metastasis.


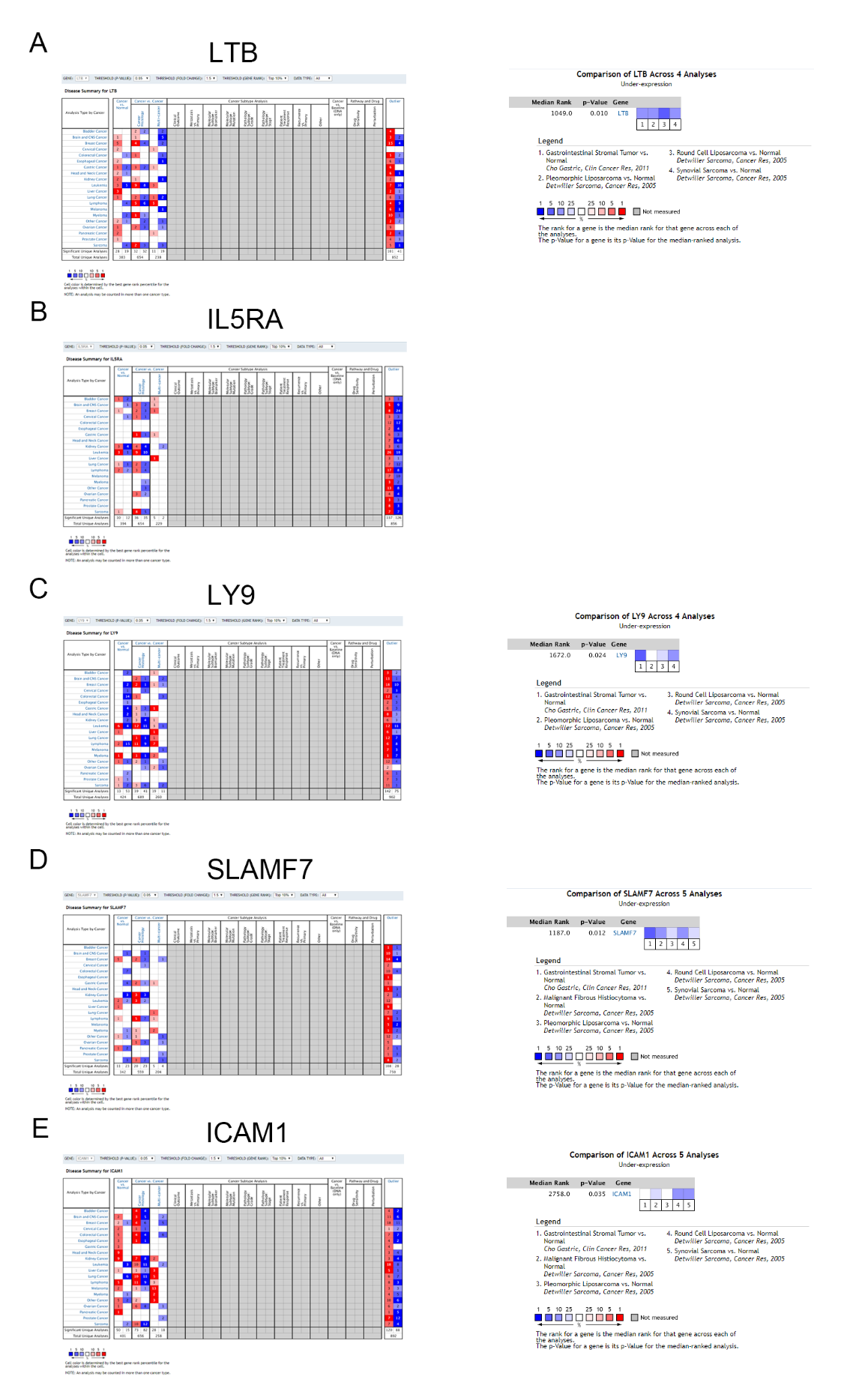


**Figure S2** Oncomine database validation. TB (A), LY9 (C), SLAMF7 (D), and ICAM1 (E) were down-regulated, while IL5RA (B) were up-regulated in different STS-related studies.


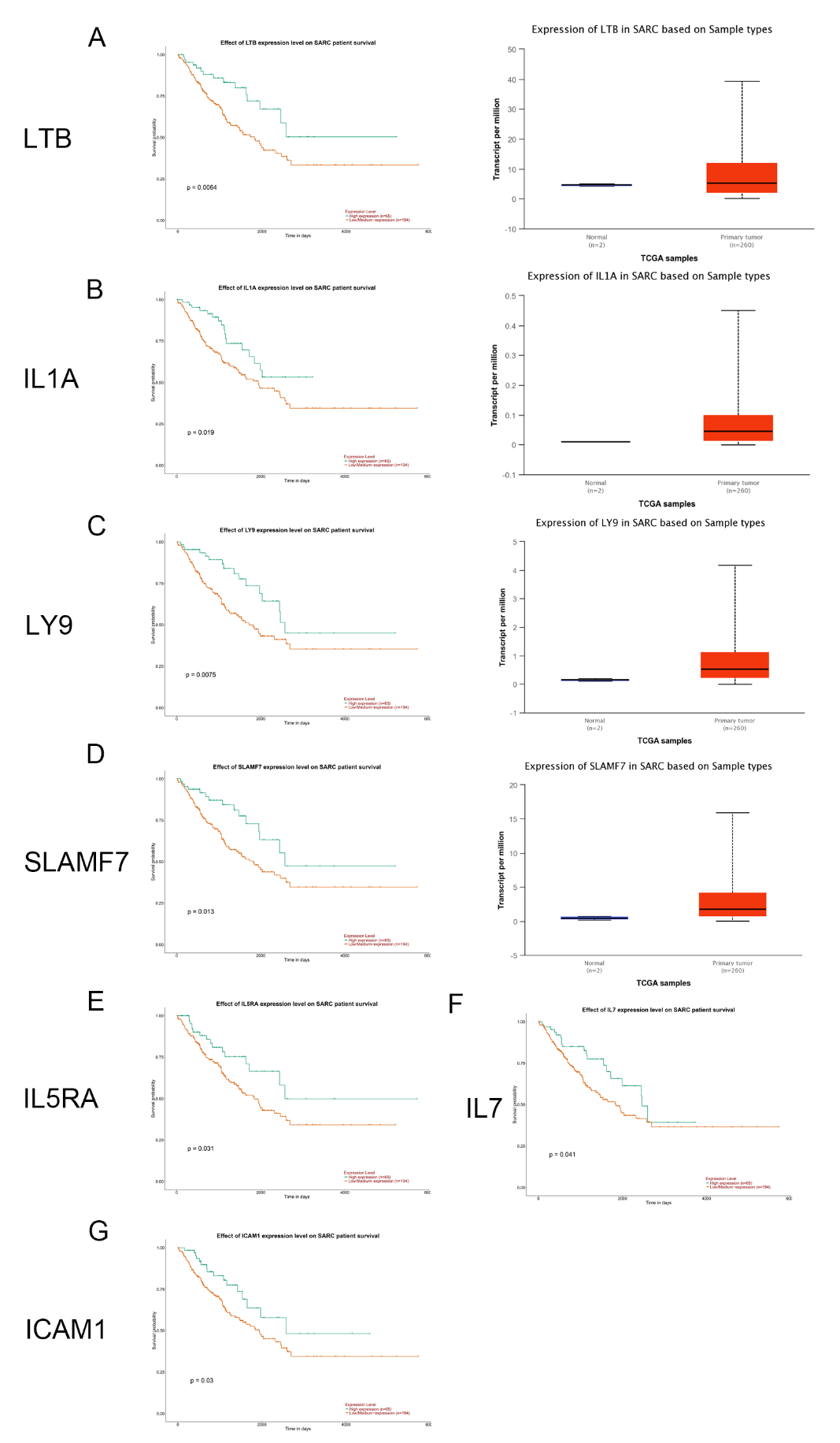


**Figure S3** UALCAN database validation. LTB (P = 0.006) (A), IL1A (P = 0.019) (B), LY9 (P = 0.008) (C), SLAMF7 (P = 0.013) (D), IL5RA (P = 0.031) (E), IL7 (P = 0.041) (F), and ICAM1 (P = 0.030) (G) were significantly correlated with STS patients’ prognosis. Besides, expression of LTB (P < 0.001) (A), IL1A (P < 0.001) (B), LY9 (P < 0.001) (C), and SLAMF7 (P < 0.001) (D) were significantly different between normal and tumor tissues.

**
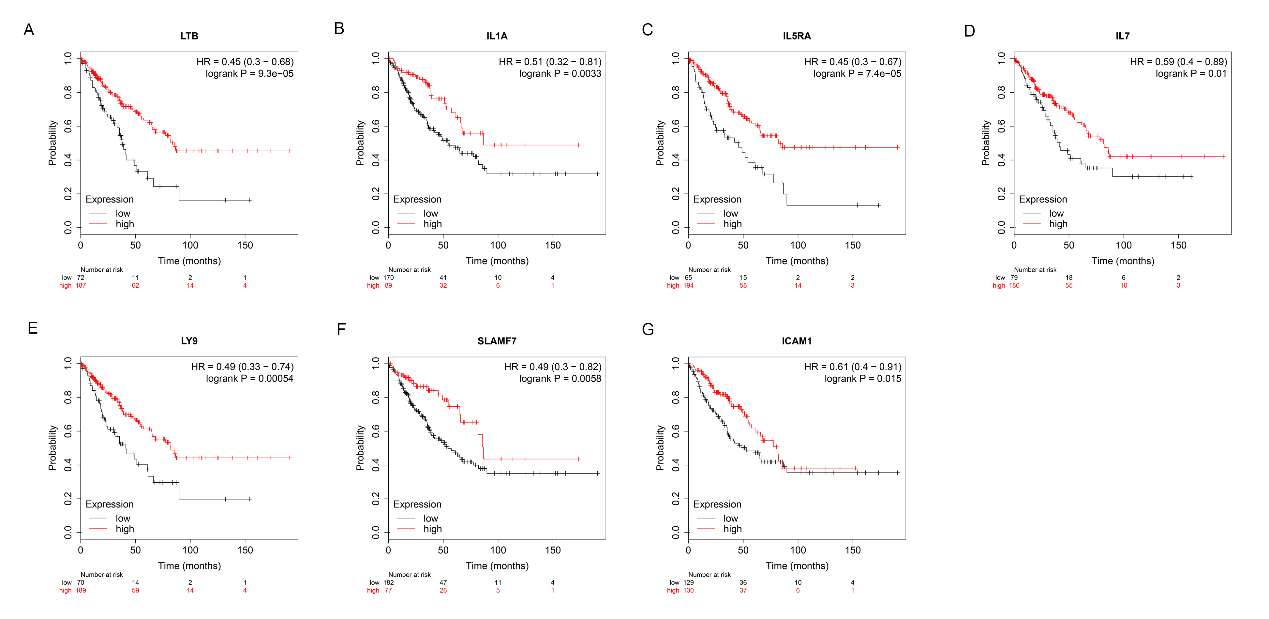
**

**Figure S4** K-M Plotter database validation. LTB (P < 0.001) (A), IL1A (P = 0.003) (B), IL5RA (P < 0.001) (C), IL7 (P = 0.041) (D), LY9 (P < 0.001) (E), SLAMF7 (P = 0.006) (F), and ICAM1 (P = 0.015) (G) were significantly correlated with patients’ prognosis.


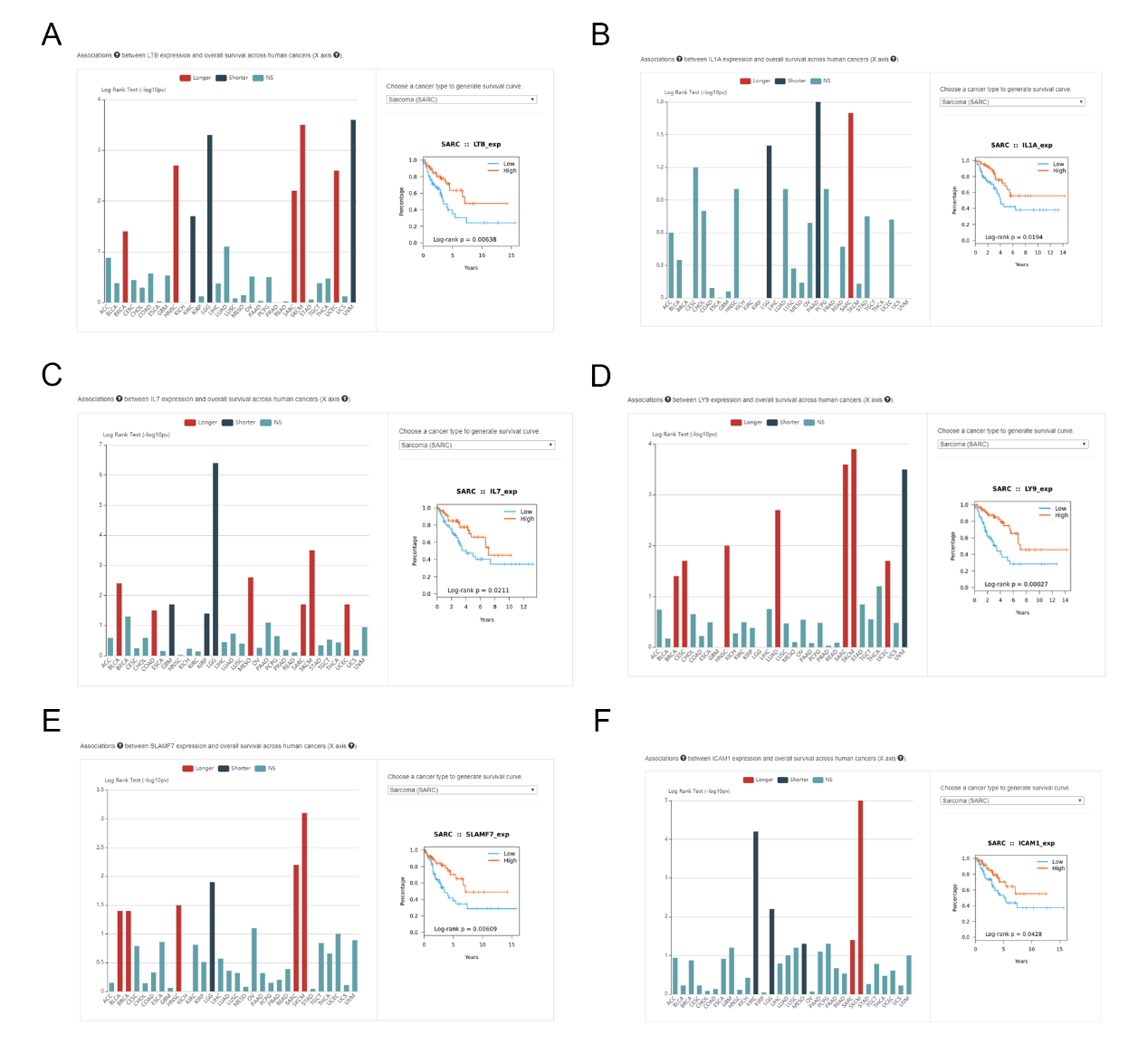


**Figure S5** TISIDB database validation. LTB (P = 0.006) (A), IL1A (P = 0.019) (B), IL7 (P = 0.021) (C), LY9 (P < 0.001) (D), SLAMF7 (P = 0.006) (E), and ICAM1 (P = 0.043) (F) were significantly correlated with patients’ prognosis.


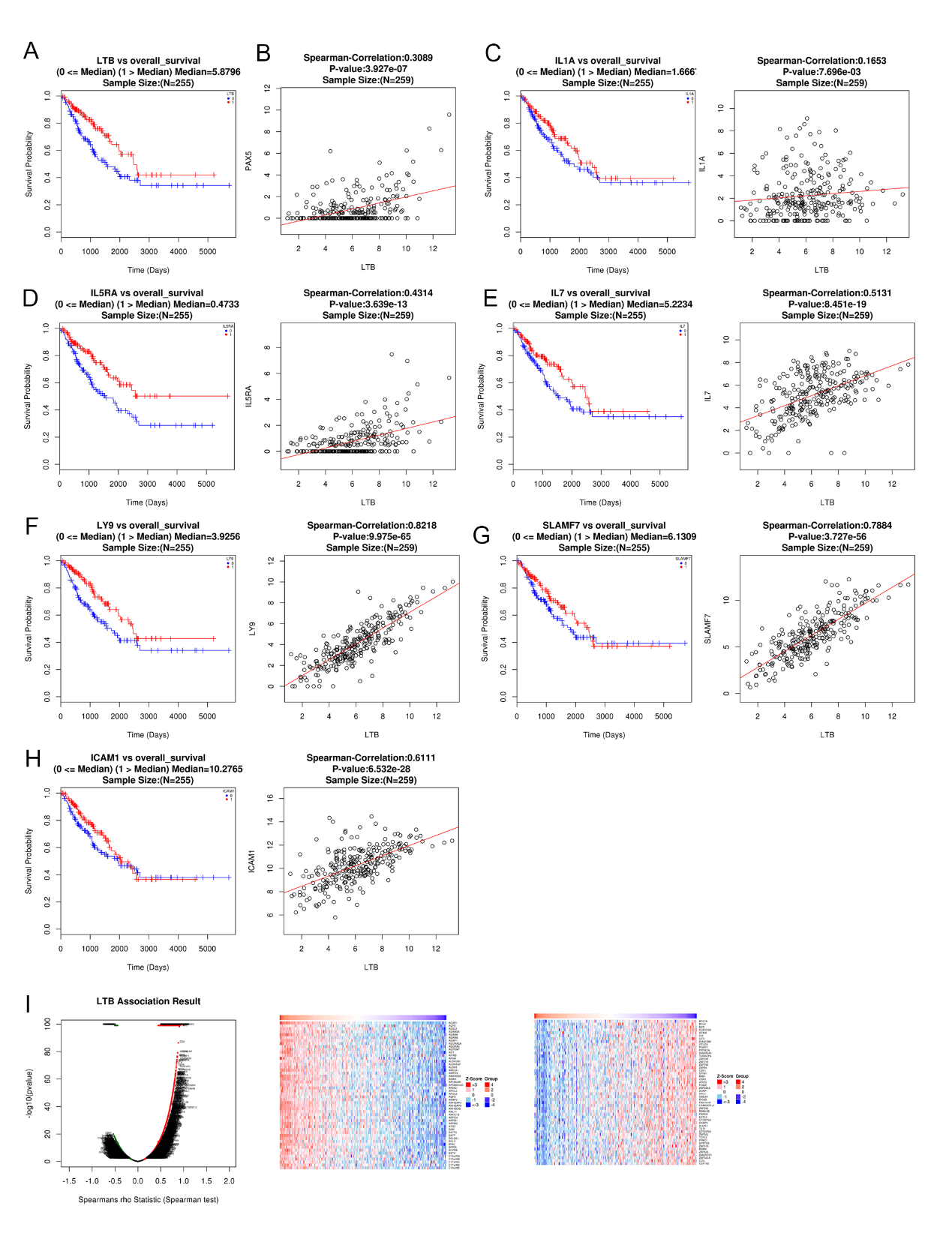


**Figure S6** LinkedOmics database validation. LTB (P < 0.001) (A), IL1A (P < 0.015) (C), IL5RA (P < 0.030) (D), IL7 (P < 0.022) (E), LY9 (P = 0.001) (F), SLAMF7 (P = 0.008) (G), and ICAM1 (P = 0.035) (H) were significantly correlated with patients’ prognosis. LTB were significantly correlated with PAX5 (P < 0.001, R = 0.31) (B), IL1A (P = 0.008, R = 0.17) (C), IL5RA (P < 0.001, R = 0.43) (D), IL7 (P < 0.001, R = 0.51) (E), LY9 (P < 0.001, R = 0.82) (F), SLAMF7 (P < 0.001, R = 0.79) (G), and ICAM1 (P < 0.001, R = 0.61) (H). (I) Volcano plot and heatmaps displayed the genes most significantly correlated with LTB.


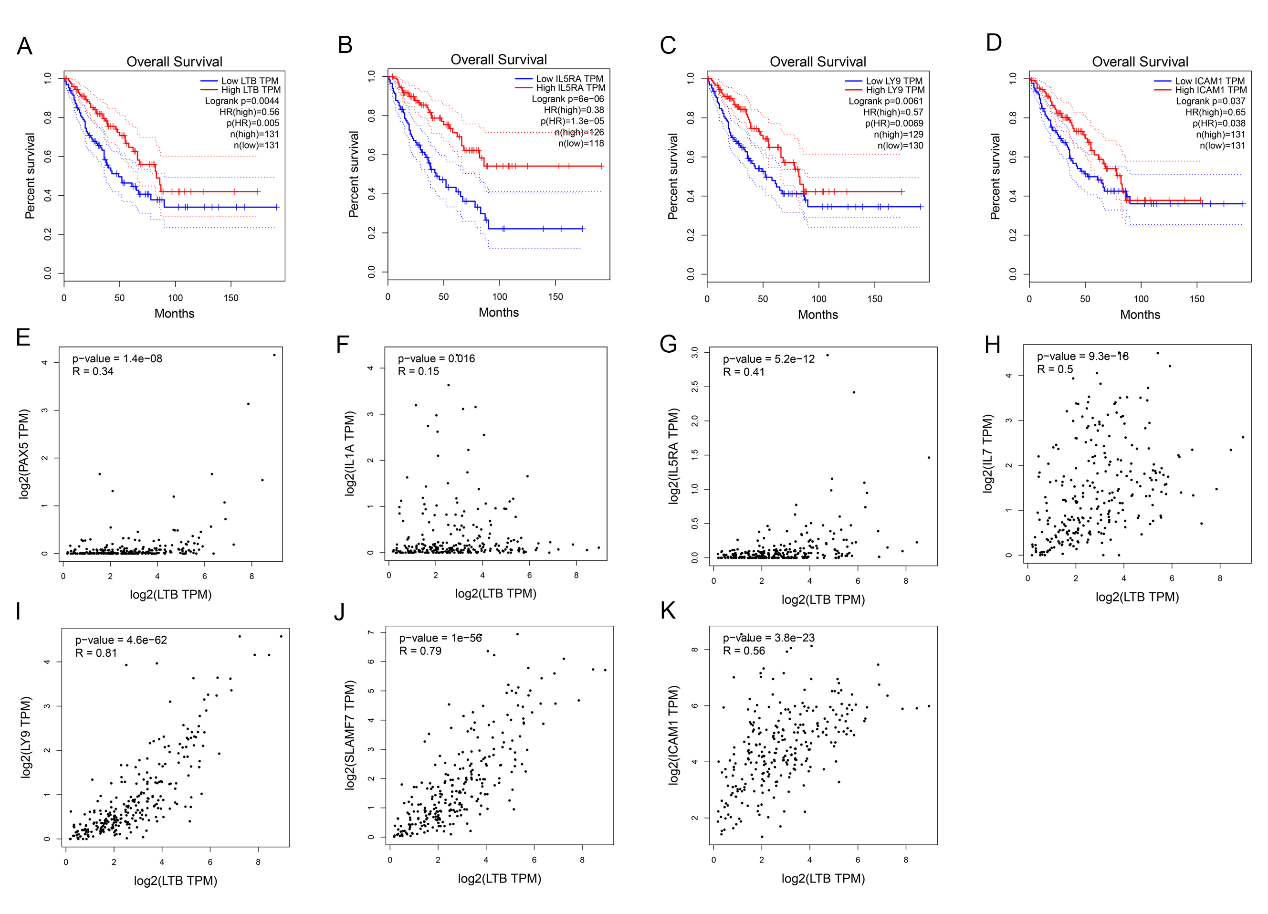


**Figure S7** GEPIA database validation. LTB (P = 0.004) (A), IL5RA (P < 0.001) (B), LY9 (P = 0.006) (C), and ICAM1 (P = 0.037) (D) were significantly correlated with prognosis. LTB was significantly correlated with PAX5 (P < 0.001, R = 0.34) (E), IL1A (P = 0.016, R = 0.15) (F), IL5RA (P < 0.001, R = 0.41) (G), IL7 (P < 0.001, R = 0.50) (H), LY9 (P < 0.001, R = 0.81) (I), SLAMF7 (P < 0.001, R = 0.79) (J), and ICAM1 (P < 0.001, R = 0.56) (K).


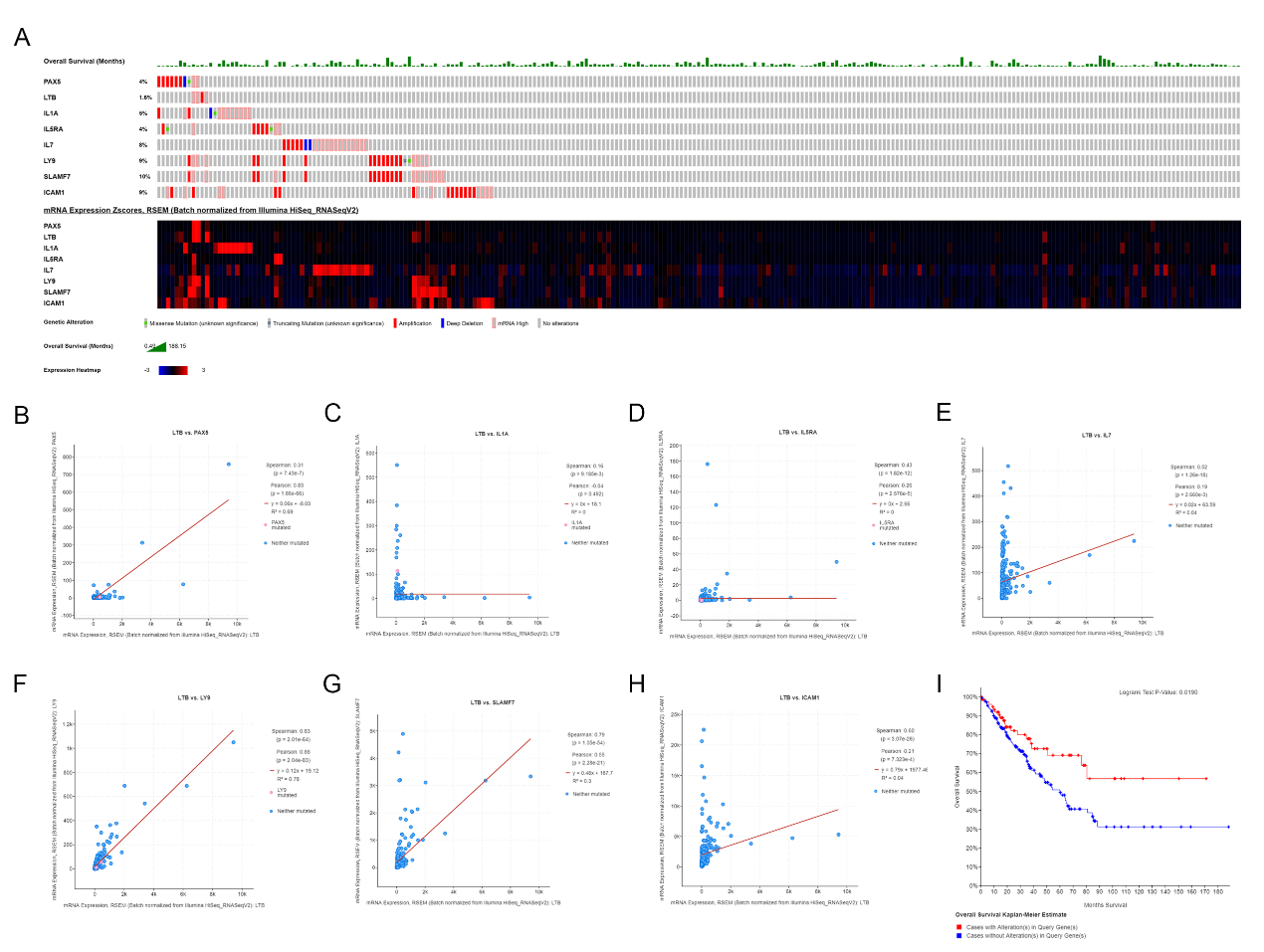


**Figure S8** cBioportal database validation. (A) mRNA expression of each biomarker illustrated by heatmap. Spearman correlation analysis shown that LTB was significantly correlated with PAX5 (P < 0.001, R = 0.31) (B), IL1A (P = 0.009, R = 0.31) (C), IL5RA (P < 0.001, R = 0.43) (D), IL7 (P < 0.001, R = 0.52) (E), LY9 (P < 0.001, R = 0.83) (F), SLAMF7 (P < 0.001, R = 0.79) (G), and ICAM1 (P < 0.001, R = 0.60) (H). (I) K-M survival analysis integrated with all the biomarkers shown that the overall expression of biomarkers was significantly related to patients’ prognosis.


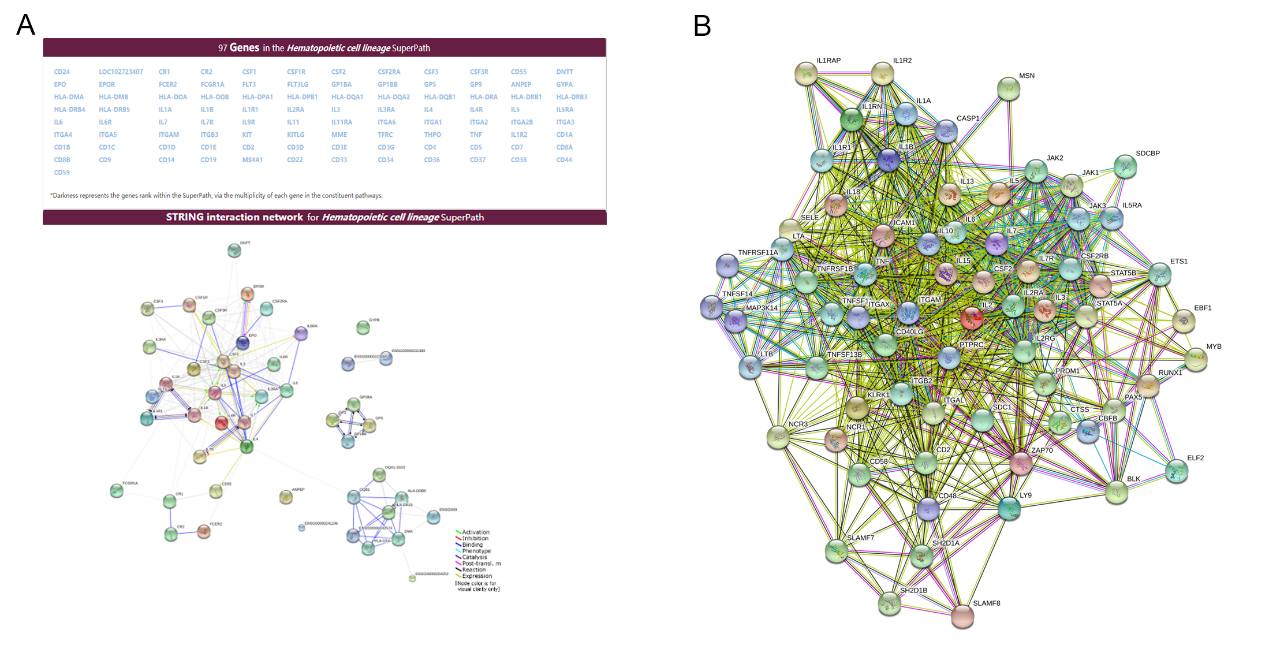


**Figure S9** Protein-protein interaction network. (A) PathCards database provided the main biomarkers actively involved in hematopoietic cell lineage pathway, including IL5RA, LY9, SLAMF7, and ICAM1. (B) STRING database shown that all the biomarkers were tightly connected with each other.
